# Magnetic Resonance Imaging and Its Clinical Correlation in Spinocerebellar Ataxia Type 3: A Systematic Review

**DOI:** 10.3389/fnins.2022.859651

**Published:** 2022-06-10

**Authors:** Kah Hui Yap, Hanani Abdul Manan, Noorazrul Yahya, Shahrul Azmin, Shahizon Azura Mohamed Mukari, Norlinah Mohamed Ibrahim

**Affiliations:** ^1^Department of Medicine, Universiti Kebangsaan Malaysia (UKM) Medical Centre, Kuala Lumpur, Malaysia; ^2^Makmal Pemprosesan Imej Kefungsian, Department of Radiology, Universiti Kebangsaan Malaysia (UKM) Medical Centre, Kuala Lumpur, Malaysia; ^3^Department of Radiology and Intervency, Hospital Pakar Kanan-Kanak, Children Specialist Hospital, Universiti Kebangsaan Malaysia (UKM), Kuala Lumpur, Malaysia; ^4^School of Diagnostic and Applied Health Sciences, Faculty of Health Sciences, National University of Malaysia, Kuala Lumpur, Malaysia

**Keywords:** spinocerebellar ataxia type 3, magnetic resonance imaging, motor, neurocognition, cerebellar-cerebral network, functional compensation

## Abstract

**Background:**

Spinocerebellar ataxia type 3 (SCA3) is a complex cerebrocerebellar disease primarily characterized by ataxia symptoms alongside motor and cognitive impairments. The heterogeneous clinical presentation of SCA3 necessitates correlations between magnetic resonance imaging (MRI) and clinical findings in reflecting progressive disease changes. At present, an attempt to systematically examine the brain-behavior relationship in SCA3, specifically, the correlation between MRI and clinical findings, is lacking.

**Objective:**

We investigated the association strength between MRI abnormality and each clinical symptom to understand the brain-behavior relationship in SCA3.

**Methods:**

We conducted a systematic review on Medline and Scopus to review studies evaluating the brain MRI profile of SCA3 using structural MRI (volumetric, voxel-based morphometry, surface analysis), magnetic resonance spectroscopy, and diffusion tensor imaging, including their correlations with clinical outcomes.

**Results:**

Of 1,767 articles identified, 29 articles met the eligibility criteria. According to the National Institutes of Health quality assessment tool for case-control studies, all articles were of excellent quality. This systematic review found that SCA3 neuropathology contributes to widespread brain degeneration, affecting the cerebellum and brainstem. The disease gradually impedes the cerebral cortex and basal ganglia in the late stages of SCA3. Most findings reported moderate correlations (*r* = 0.30–0.49) between MRI features in several regions and clinical findings. Regardless of the MRI techniques, most studies focused on the brainstem and cerebellum.

**Conclusions:**

Clinical findings suggest that rather than individual brain regions, the connectivity between different brain regions in distributed networks (i.e., cerebellar-cerebral network) may be responsible for motor and neurocognitive function in SCA3. This review highlights the importance of evaluating the progressive changes of the cerebellar-cerebral networks in SCA3 patients, specifically the functional connectivity. Given the relative lack of knowledge about functional connectivity on SCA3, future studies should investigate possible functional connectivity abnormalities in SCA3 using fMRI.

## Introduction

Spinocerebellar ataxia type 3 (SCA3) is the most common dominantly inherited ataxia worldwide (Paulson, 2012). SCA3 neuropathology involves mutation of cytosine-adenine-guanine (CAG) repeat in the *ATXN3* gene that codes for the *ataxin-3* protein. This elongated polyglutamine tract formed protein aggregates primarily deposited and resulted in cerebellar cell deaths (Pedroso et al., 2013). As a result, SCA3 is primarily characterized by progressive cerebellar degeneration, resulting in increasingly worsening ataxia symptoms, including gait abnormalities, dysarthria, and abnormal eye movements (Bodranghien et al., 2016). Ataxia severity is commonly measured using the Scale for the Assessment and Rating of Ataxia (SARA; 0–40) and the International Cooperative Ataxia Rating Scale (ICARS; 0–100) (Zhou et al., 2011). Apart from the cerebellum, individual differences in the extracerebellar involvement further contribute to the varying degree of ataxia, non-ataxia, and cognitive impairment in SCA3 (Lindsay and Storey, 2017). In addition, the severity of ataxia may exacerbate the cognitive impairment on pen-and-paper tests (Yap et al., 2022b). Therefore, clinical measures alone might not reflect progressive disease changes, requiring correlations with objective measures such as magnetic resonance imaging (MRI) (Döhlinger et al., 2008).

Magnetic resonance imaging has many advantages, including high spatial resolution, and is capable of morphological and functional imaging. Advanced MRI techniques are able to assess structural and volumetric changes, metabolic alterations (magnetic resonance spectroscopy; MRS), white matter integrity (diffusion-tensor imaging; DTI), and functional abnormalities (task-based and resting-state functional MRI; fMRI) (Currie et al., 2013). These neuroimages may provide biologically-relevant measures that may be valuable biomarkers for spinocerebellar ataxia (SCA) (Adanyeguh et al., 2018).

MRI structural abnormalities have been widely reported in SCA3 and correlations with clinical findings have been explored (Wan et al., 2020). However, functional connectivity abnormalities have been poorly assessed and there is a relative lack of knowledge about fMRI application on SCA3. A recent review has discussed the relationship between MRI structural abnormalities, disease severity, CAG repeats, and disease duration qualitatively (Wan et al., 2020). However, an attempt to systematically examine the association between brain regions and clinical scales is lacking (Wan et al., 2020). Therefore, understanding the association strength between MRI abnormality and each clinical symptom will help understand the brain-behavior relationship in SCA3. We systematically reviewed published articles that have evaluated the brain MRI profile of SCA3, including studies that reported correlations with clinical outcomes. We aimed to identify brain regions responsible for motor and cognitive decline in SCA3, respectively.

## Methods

### Search Strategy

We performed a literature search using two electronic databases: Medline (Ovid) and Scopus. The search identified articles providing information on the brain MRI profile of SCA3 with or without comparisons against healthy comparisons (HC). We included only original articles written in English and reported human studies. To avoid selection bias, we excluded articles with fewer than 12 participants in each group (i.e., SCA3 vs. HC) (Szucs and Ioannidis, 2020). We also excluded studies that used MRI with <1.5 Tesla field (T) to minimize the risk of Type II error. We considered 1.5 and 3.0 T MRI as equal there was little difference in the diagnostic accuracy (Wardlaw et al., 2012). We searched the articles on March 23, 2021, using Medical Subject Headings (MeSH) terms such as “SCA3” and “Machado-Joseph disease,” coupled with terms related to MRI, such as “volumetric,” “morphometric,” “functional,” “resting-state,” “magnetic resonance,” “spectroscopy” “diffusion tensor imaging,” “tractography,” “MRI,” “DTI,” “white matter,” and “gray matter.”

### Data Extraction and Analysis

**Figure 1:**
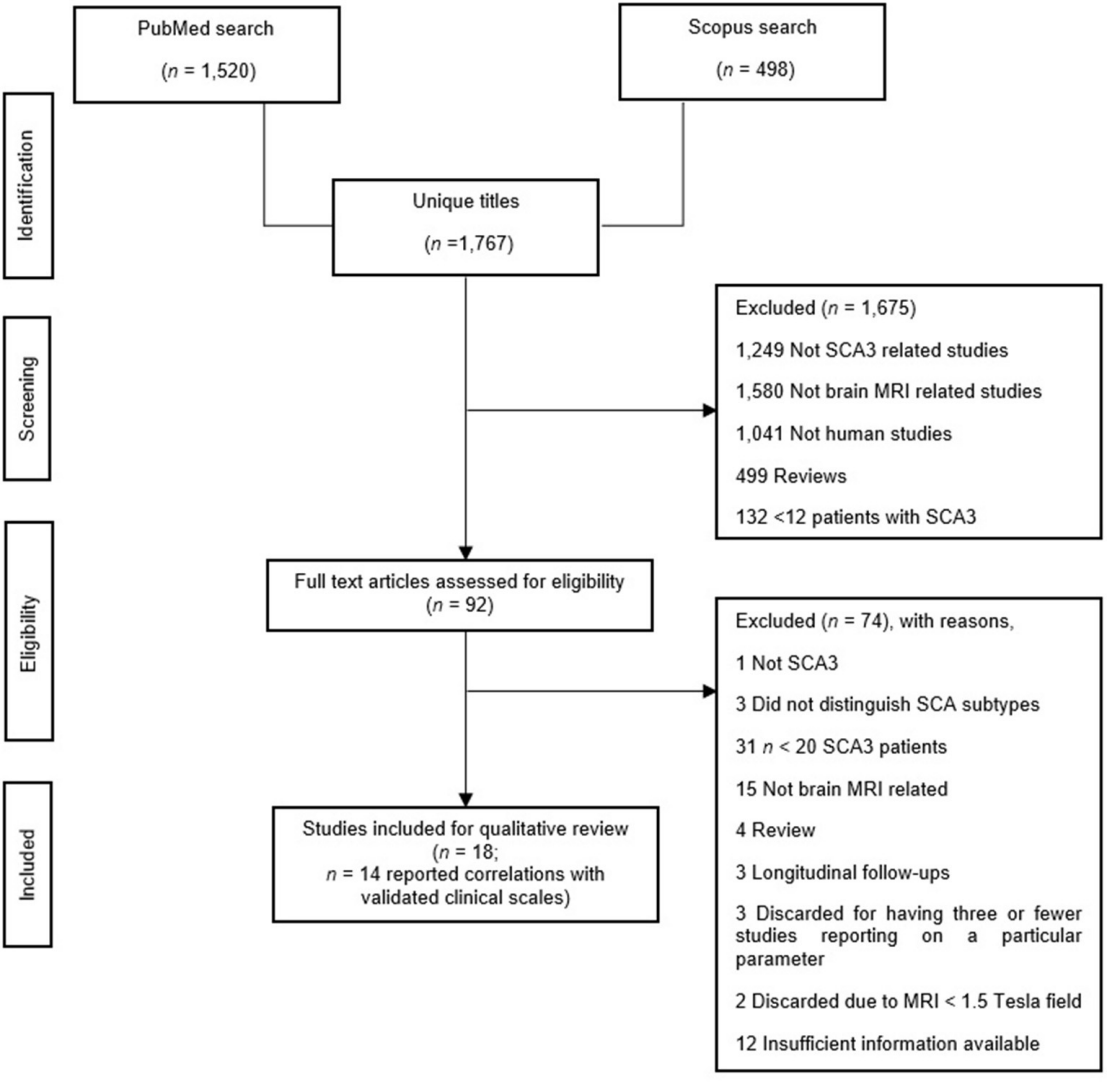
Study search in accordance with the PRISMA guideline (Page et al., 2021).

The first author performed an initial eligibility screening by going through the titles and abstracts of 1,767 initially identified articles, then cross-checked by another author. As illustrated in Figure 1, we adhered to the Preferred Reporting Items for Systematic Review and Meta-Analyses (PRISMA) reporting guidelines (Page et al., 2021). Finally, we included a total of 29 articles in this review. Twenty papers examined structural MRI [volumetric: *n* = 12 (Bürk et al., 1996; Etchebehere et al., 2001; Yoshizawa et al., 2003; Liang et al., 2009; Schulz et al., 2010; Camargos et al., 2011; D'Abreu et al., 2011; Ogawa et al., 2012; De Rezende et al., 2015; Nunes et al., 2015; Rezende et al., 2018; Jao et al., 2019b), voxel-based morphometry (VBM): *n* = 8 (Schulz et al., 2010; D'Abreu et al., 2012; Guimarães et al., 2013; Lopes et al., 2013; Kang et al., 2014; Hernandez-Castillo et al., 2017; Peng et al., 2019; Guo et al., 2020), and surface analysis: *n* = 4 (De Rezende et al., 2015; Nunes et al., 2015; Rezende et al., 2018; Arruda et al., 2020)], seven papers examined MRS (Lei et al., 2011; Lirng et al., 2012; Wang et al., 2012; Lopes et al., 2013; Chen et al., 2014; Adanyeguh et al., 2015; Peng et al., 2019), and ten papers examined DTI (Guimarães et al., 2013; Lopes et al., 2013; Kang et al., 2014; Nunes et al., 2015; Wu et al., 2017; Rezende et al., 2018; Jao et al., 2019a; Peng et al., 2019; Meira et al., 2020; Inada et al., 2021). We summarized findings in Supplementary Table 3 and outlined the details in Supplementary Table 2. Subsequently, we synthesized the findings into two parts: (1) Difference in MRI-related findings between SCA3 and HC, and (2) MRI-related findings and their correlation with clinical scales in SCA3, specifically, ataxia, non-ataxia, and cognitive function. Due to the lack of findings, that is, three or fewer articles, we only discussed these findings concerning avenues for future studies, if applicable: 3D fractal dimension (3D-FD) analysis: *n* = 3 (Huang et al., 2017; Jao et al., 2019b; Wang et al., 2020), texture analysis: *n* = 1 (De Oliveira et al., 2010), relaxometry: *n* = 1 (Guimarães et al., 2013), and neuromelanin contrast: *n* = 1 (Nakata et al., 2020), and task-based fMRI (Duarte et al., 2016).

As per the National Health Medical Research Council guidelines (National Health Medical Research Council, 2009), we classified all 29 studies as Level III-2 evidence. We adapted the National Institutes of Health quality assessment tool for case-control studies (National Institutes of Health, 2019). The results, displayed in Supplementary Table 1, showed that all studies were of excellent quality due to the consistent use of standardized diagnostic criteria in screening SCA3 patients and HC.

In the present review, we were interested in the outcomes of validated clinical scales, including the SARA and ICARS for ataxia severity and well-established neuropsychological tests for cognitive impairment. We did not consider outcomes derived from subscales from SARA and ICARS. In addition, screening tests usually lack validation and may be vulnerable to ceiling effects, making them relatively insensitive when used in isolation. Such screening tests included Mini-Mental State Examination (Franco-Marina et al., 2010) and the Montreal Cognitive Assessment (Smith et al., 2007).

The correlation coefficient, *r*, was recorded as the correlation between the clinical and MRI outcomes in SCA3. The *r* values of 0.10, 0.30, and 0.50 indicate small, moderate, and strong correlations, respectively (Cohen, 2013). In this review, a larger *r* indicated a stronger correlation between clinical and MRI outcomes in SCA3 (Cohen, 2013). Given the nature of the tests (e.g., higher scores in SARA indicate greater severity, whereas higher scores in ICARS indicate a lower severity), we only considered the absolute values. We did not consider studies that reported correlation without providing the *r* values.

## Results

### SCA3 vs. Healthy Comparisons

#### Structural MRI

##### Cerebrum (Cortical)

Two studies analyzed the volumetric changes in the cortical regions of SCA3 patients (Schulz et al., 2010; Jao et al., 2019b). Both studies reported atrophy in the temporal lobe. While the first study reported atrophy in the frontal, parietal, and occipital lobes (Jao et al., 2019b), the second study reported non-significant increased volume in these regions (Schulz et al., 2010).

Six studies analyzed the VBM changes in the cortical regions' gray matter volume (GMV) (Schulz et al., 2010; D'Abreu et al., 2012; Lopes et al., 2013; Hernandez-Castillo et al., 2017; Peng et al., 2019; Guo et al., 2020). While a study reported no change in the overall GMV in the cerebral hemisphere (Schulz et al., 2010), reduced GMV were reported separately in cerebral lobes, including frontal (precentral gyrus, and superior, medial, and inferior frontal gyri), parietal (postcentral gyrus, superior, and inferior parietal lobes, and association cortex), temporal (insula, fusiform gyrus, and middle, and superior temporal gyri), and occipital (lingual gyrus, cuneus, and occipital gyri) lobes. Lack of change in the GMV of the overall cerebral hemispheres may suggest that individual difference in the SCA3 neuropathology differentially affects the cortical regions, which may explain heterogeneous clinical manifestation across SCA3 patients (Pedroso et al., 2013). Alternatively, using a small sample size (*n*SCA3 = 24 vs. *n*HC = 31) and four different scanners may contribute to the methodological limitation that may explain the null finding (Schulz et al., 2010).

Four studies analyzed the surface of the cortical regions (De Rezende et al., 2015; Nunes et al., 2015; Rezende et al., 2018; Arruda et al., 2020). These studies reported reduced cortical thickness in the frontal (precentral and superior frontal gyri), temporal (superior temporal gyrus), and occipital lobes. While surface analysis selectively investigates cortical thickness, VBM provides a composite measure of GMV, including cortical surface area or cortical folding, as well as cortical thickness (Hutton et al., 2009). This difference between VBM and surface analysis may explain the inconsistent findings regarding the GMV of the parietal lobe; that is, reduced cortical folding and surface area contributes to the GMV loss in the parietal lobe. Conversely, a relatively preserved cortical thickness in the parietal lobe may only be affected later in the SCA3 pathology.

##### Cerebrum (Subcortical)

Five studies analyzed the volumetric changes in the subcortical regions of SCA3 patients (D'Abreu et al., 2011; De Rezende et al., 2015; Nunes et al., 2015; Rezende et al., 2018; Jao et al., 2019b). These studies reported atrophy in the diencephalon (ventral diencephalon and thalamus), limbic system (limbic lobe and hippocampus), basal ganglia (globus pallidus, putamen, caudate, and substantia nigra), and lenticular fasciculus.

Six studies analyzed the VBM changes in the GMV of subcortical regions (Schulz et al., 2010; D'Abreu et al., 2012; Guimarães et al., 2013; Lopes et al., 2013; Kang et al., 2014; Guo et al., 2020). All studies consistently reported reduced GMV in the limbic system (limbic lobe and posterior cingulate), and claustrum. Three studies reported reduced GMV in the basal ganglia (lentiform nucleus, caudate, and putamen), absent in the remaining study characterized by a smaller sample size and the use of four different scanners (Schulz et al., 2010).

Only one study analyzed and reported reduced cortical thickness of the limbic system (posterior cingulate) (Rezende et al., 2018). This scarcity in research is likely because while theory and convention informed the classification of the limbic system under the subcortical region (Berkowitz, 2016), the limbic system involves cortical structures (Rolls, 2015). This classification may explain the scarcity of surface analysis on the limbic system.

##### Brainstem

Nine studies analyzed the volumetric changes in the brainstem of SCA3 patients (Bürk et al., 1996; Yoshizawa et al., 2003; Liang et al., 2009; Schulz et al., 2010; Camargos et al., 2011; Ogawa et al., 2012; De Rezende et al., 2015; Nunes et al., 2015; Rezende et al., 2018). These studies reported widespread atrophy in the overall brainstem, including pons, midbrain, and medulla. Two studies reported atrophy in the midbrain, while one study did not show any changes in the aforementioned brain regions (Liang et al., 2009). While the sample size may not explain the discrepancy in findings, the difference in the brain structure across ethnicity may be contributory (i.e., East Asian vs. Western) (Tang et al., 2018).

Six studies analyzed the VBM changes in the GMV of the brainstem (Schulz et al., 2010; D'Abreu et al., 2012; Guimarães et al., 2013; Kang et al., 2014; Hernandez-Castillo et al., 2017; Peng et al., 2019). These studies consistently reported reduced GMV in the overall brainstem, including medulla, midbrain, and pyramids. Conversely, three studies reported reduced GMV in the pons, while one did not. A smaller sample size may explain the absence of reduced GMV in the pons (Schulz et al., 2010). Similar to the GMV in the brainstem, the overall brainstem also showed reduced WBV, including medulla, pyramids, pons, and midbrain.

##### Cerebellum

Ten studies analyzed the volumetric changes in the cerebellum of SCA3 patients (Bürk et al., 1996; Etchebehere et al., 2001; Liang et al., 2009; Schulz et al., 2010; Camargos et al., 2011; Ogawa et al., 2012; De Rezende et al., 2015; Nunes et al., 2015; Rezende et al., 2018; Jao et al., 2019b). These studies consistently reported atrophy in the overall cerebellum, including cerebellar hemispheres and vermis.

Eight studies analyzed the VBM changes in the GMV of the cerebellum (Schulz et al., 2010; D'Abreu et al., 2012; Guimarães et al., 2013; Lopes et al., 2013; Kang et al., 2014; Hernandez-Castillo et al., 2017; Peng et al., 2019; Guo et al., 2020). These studies consistently reported reduced GMV in the cerebellum, including cerebellar hemispheres (cerebellar hemispheres, tonsil, and inferior semilunar lobule) and vermis (overall, culmen, declive, uvula, fastigium, and tuber). On the other hand, two studies analyzed the white matter volume (WMV) (Schulz et al., 2010; Guimarães et al., 2013). These studies reported reduced WMV in the overall cerebellum, including left cerebellar hemisphere, vermis (tonsil, dentate, uvula, fastigium, and tuber, sparing the nodule), and cerebellar peduncles.

##### Summary

SCA3 neuropathology involves widespread atrophy across the brain, including cortical and subcortical cerebral regions, brainstem, and cerebellum. The present findings suggest that the volumetric loss in the cerebrum involved both GMV loss and cortical thinning. On the other hand, findings on the brainstem and cerebellum implicated that the volumetric loss in these regions is not restricted to GMV loss; WMV loss is similarly prominent in these regions (Schulz et al., 2010; Guimarães et al., 2013). Future studies may investigate the difference between GMV and WMV loss in the cerebrum, contributing to the heterogeneous clinical profile in SCA3 patients.

#### MRS

The levels of N-acetyl aspartate (NAA), glutamate (Glu), Choline (Cho), myo-Inositol (myo-Ins), and Creatinine (Cr) are the primary metabolites being studied. NAA and Glu concentration reflects the neuronal viability and integrity, in which reduced concentrations indicate neuronal and axonal injury or loss (Moffett et al., 2007). Cho is a precursor of acetylcholine and is essential for cell membrane and neurotransmitter metabolism. Myo-Ins is a biomarker for glial activation in response to neuronal injury and degeneration (Duarte et al., 2012). Cr reflects brain energy metabolism and is relatively stable under normal conditions, serving as a reference for comparison (Lirng et al., 2012).

##### Cerebrum (Subcortical)

Only one study analyzed and reported reduced NAA/Cr and NAA/Cho in the diencephalon (thalamus) of SCA3 patients (Peng et al., 2019). The same study reported no change of NAA/Cr in the basal ganglia (putamen) (Peng et al., 2019).

##### Brainstem

Only one study reported reduced NAA and glutamate (Glu) and increased Cr and myo-Ins in the brainstem (pons) (Adanyeguh et al., 2015). The finding suggests that changes in NAA/Cr ratio in the pons may represent a better early biomarker for SCA3 neuropathology (Adanyeguh et al., 2015; Peng et al., 2019). Consistently, changes in the pons, alongside the cerebellum, are promising biomarkers for the disease progression in SCA3 (Adanyeguh et al., 2018).

##### Cerebellum

Six studies analyzed the metabolic changes in the cerebellum of SCA3 patients (Lei et al., 2011; Lirng et al., 2012; Wang et al., 2012; Chen et al., 2014; Adanyeguh et al., 2015; Peng et al., 2019). One study reported reduced NAA and Glu and increased Cr and myo-Ins in the vermis (Adanyeguh et al., 2015). The remaining studies consistently reported reduced NAA/Cr in the cerebellar hemispheres, vermis (overall and dentate), and middle cerebellar peduncles. Four studies reported reduced NAA/Cho in the cerebellar hemispheres, vermis (overall and dentate), and middle cerebellar peduncles.

Conversely, while four studies reported no change in the Cho/Cr in the overall cerebellum (Lei et al., 2011; Lirng et al., 2012; Wang et al., 2012; Chen et al., 2014), a study reported reduced Cho/Cr in the vermis (Peng et al., 2019). This finding suggests that the SCA3 neuropathology may affect the vermis in the early stage of the disease. The vermis is typically more affected than the cerebellar hemispheres in genetic disorders (Aldinger and Doherty, 2016), although this finding has not been replicated in the SCA3 population.

A study reported that the following metabolites were reduced in the cerebellum: NAA/Total Cr (Cr+PCr) ratio, NAA+N-acetyl-aspartyl-glutamate (NAA+NAAG)/Cr+PCr ratio, and glutamine (Glx)/Cr+PCr ratio (Lopes et al., 2013). The same study also reported that the following metabolites did not change in the cerebellum: Glu/Cr+PCr ratio, phosphorylcholine (PCh)/Cr+PCr ratio, and myo-Ins/Cr+PCr ratio (Lopes et al., 2013).

##### Summary

While far from being a sensitive biomarker, MRS findings may explain the metabolic changes and neuronal dysfunction before the neuronal loss and clinical manifestation of SCA3 (Chen et al., 2014). Reduced NAA and Glu primarily present in the pons and vermis of SCA3 patients imply reduced metabolism and neuronal loss in these regions (Adanyeguh et al., 2015). Conversely, increased myo-Ins is likely a response to the degenerative nature of SCA3, while increased Cr may be a compensatory response to maintain the energy supply of brain tissue (Brewer and Wallimann, 2000). The lack of studies on the absolute concentration of individual metabolites compared to metabolite ratios is likely because the latter offers a more robust approach in accounting for a small sample size (Hoch et al., 2017).

Overall, reduced metabolism primarily involves the brainstem (pons) and cerebellum (vermis). These findings may represent an early biomarker of the SCA3 neuropathology that precedes structural loss. Compared to its structural counterpart, MRS studies using individual metabolites on the cerebrum of SCA3 are lacking. Likewise, several metabolite ratios were preliminary and deserved further study (Lopes et al., 2013).

#### DTI

We only considered fractional anisotropy (FA) to represent a reliable marker for DTI changes (Bennett et al., 2010). Fractional anisotropy (FA) is sensitive to microstructural changes, reflecting white matter fiber density, axonal diameter, and myelination. A higher FA indicates greater white matter integrity and vice versa (Dong et al., 2012).

##### Cerebrum (Cortical)

Four studies reported reduced FA in the frontal (precentral and middle frontal gyri), parietal (postcentral gyrus temporal), and occipital lobes (Kang et al., 2014; Rezende et al., 2018; Jao et al., 2019a; Inada et al., 2021).

##### Cerebrum (Subcortical)

Five studies reported reduced FA in the corona radiata, internal and external capsules, thalamic radiation, forceps, cingulate fasciculus, parietal-temporal superior and inferior longitudinal fasciculus, and corpus callosum (Kang et al., 2014; Rezende et al., 2018; Jao et al., 2019a; Meira et al., 2020; Inada et al., 2021).

##### Brainstem

Eight studies analyzed the FA changes in the brainstem (Guimarães et al., 2013; Lopes et al., 2013; Kang et al., 2014; Wu et al., 2017; Rezende et al., 2018; Jao et al., 2019a; Meira et al., 2020; Inada et al., 2021). These studies reported reduced FA in the brainstem (overall and pons). Regarding the tracts, studies reported reduced FA in the cortical-spinal tract, lemniscus, and pons crossing tract.

##### Cerebellum

Seven studies analyzed the FA changes in the cerebellum (Guimarães et al., 2013; Kang et al., 2014; Wu et al., 2017; Rezende et al., 2018; Jao et al., 2019a; Peng et al., 2019; Inada et al., 2021). A study reported reduced FA in the cerebellar hemispheres and vermis (nodule, culmen, dentate, fastigium, and lingual) (Guimarães et al., 2013). Regarding the tracts, studies reported reduced FA in the cerebellar peduncles (overall, superior, middle, and inferior cerebellar peduncles).

##### Summary

DTI studies reported a widespread decrease in FA in the white matter across the whole brain, with relatively more studies in the cerebellum and the brainstem. These studies also reported white matter abnormalities in several fiber pathways. These fibers typically involved cerebellar connecting tracts, including cerebellar peduncles, thalamic radiations, and cortical-spinal tract. These findings suggest that apart from individual brain regions that are impaired, disrupted connectivity between cerebellum from other brain regions is equally contributory to the clinical manifestations in SCA3.

### MRI vs. Clinical Correlations in SCA3

#### Motor (Ataxia)

##### Structural MRI

###### Cerebrum (Cortical).

A study reported that ataxia severity using SARA moderately correlates with GMV of frontal (precentral gyrus: *r* = −0.30, middle frontal cortex: *r* = −0.33, paracentral lobule: *r* = −0.31), and temporal (transverse temporal gyrus: *r* = −0.30, superior temporal sulcus: *r* = −0.35, transverse temporal cortex: *r* = −0.35) lobes (De Rezende et al., 2015). Another study showed that ataxia severity using ICARS moderately correlates with the GMV of occipital (*r* = 0.44) lobes, and strongly correlates with the GMV of the frontal (*r* = 0.62), parietal (*r* = 0.57), temporal (*r* = 0.72) lobes (D'Abreu et al., 2012). The findings showed that the correlations were more consistent using SARA; the *r* ranged between −0.35 and −0.30. Before attributing the findings to the differences in clinical scales, it is important to rule out potential confounding factors. Specifically, one study correlates SARA scores with specific regions (e.g., gyrus) (De Rezende et al., 2015), whereas another study correlates ICARS with the entire lobe (D'Abreu et al., 2012).

###### Cerebrum (Subcortical).

Two studies reported correlations between changes in the subcortical regions and ataxia severity using SARA (Schulz et al., 2010; De Rezende et al., 2015). A study reported that ataxia severity moderately correlates with the volumetric changes in the dorsal striatum (*r* = −0.36); the correlation was marginally stronger when only the caudate nucleus was taken into account (*r* = −0.46) (Schulz et al., 2010). Another study reported strong correlations between SARA and the volumetric changes in the diencephalon (right: *r* = 0.58, left: *r* = 0.64, thalamus: *r* = 0.62) (De Rezende et al., 2015). Consistent with the findings, the caudate nucleus and thalamus are among the subcortical regions most affected in SCA3 (Meira et al., 2020). Degeneration in the thalamus may contribute to the severity of ataxia (Rüb et al., 2003). On the other hand, striatal degeneration may result in parkinsonism in SCA3 (Nunes et al., 2015), confounding the SARA scores.

###### Brainstem.

Ataxia severity using SARA moderately correlates with the volumetric changes in the midbrain (*r* = −0.47) and medulla (*r* = −0.48), and strongly correlates with the volumetric changes in the total brainstem (*r* = −0.58 ~ −0.68) (Schulz et al., 2010; De Rezende et al., 2015), and pons (*r* = −0.56) (Schulz et al., 2010). Similarly, ICARS strongly correlates with volumetric changes in the total brainstem (*r* = −0.62), midbrain (*r* = −0.53) and pons (*r* = −0.57 ~ −0.68) (Liang et al., 2009; Camargos et al., 2011).

###### Cerebellum.

Ataxia severity using SARA moderately correlates with the volumetric changes in the total cerebellum (*r* = −0.42 ~ −0.45) (Liang et al., 2009; Schulz et al., 2010) and cerebellar hemispheres (*r* = −0.46) (Schulz et al., 2010). Similarly, ataxia severity using ICARS moderately correlates with the volumetric changes in the right hemisphere (*r* = −0.49), while strongly correlates with the volumetric changes in the total cerebellum (*r* = −0.60), left hemisphere (*r* = −0.63), and vermis (*r* = −0.52) (Camargos et al., 2011). Likewise, ataxia severity using ICARS strongly correlates with the GMV of the total cerebellum (*r* = 0.73) (D'Abreu et al., 2012). Lastly, a study showed that the SARA moderately correlates with the WMV of the cerebellum (*r* = −0.46) (Arruda et al., 2020). The discrepancy in the *r* suggests that GMV of the total cerebellum may represent a more sensitive biomarker than volumetric changes and WMV for ataxia severity in SCA3.

###### Summary.

Overall, the findings are consistent with a previous finding that both SARA and ICARS are similarly reliable and effective in assessing ataxia severity in SCA3 (Zhou et al., 2011). More studies, exploring correlations between SARA and ICARS and MRI abnormalities, are needed to confirm the findings. Nevertheless, the results are consistent with the body of evidence that structural loss in the cerebellum and brainstem, especially the GMV, contribute toward ataxia severity in SCA3 (Eichler et al., 2011). Thalamus atrophy may exacerbate ataxia severity in SCA3 (Rüb et al., 2003). Interestingly, ataxia severity in SCA3 was also associated with cortical atrophy, to which further investigations are needed to examine its role in ataxia.

##### MRS

###### Brainstem.

Ataxia severity using SARA strongly correlates with NAA (*r* = −0.82), Cr (*r* = 0.64), and myo-Ins (*r* = 0.69) in the brainstem (Adanyeguh et al., 2015).

###### Cerebellum.

Ataxia severity using SARA showed no correlation between SARA and NAA/Cr ratio in the total cerebellum (Wang et al., 2012; Adanyeguh et al., 2015). However, when examining specific cerebellar regions, SARA moderately correlates with the NAA/Cr ratio in the dentate nucleus (*r* = 0.45) and strongly correlates with the middle cerebellar peduncle (*r* = 0.95) (Lei et al., 2011). Ataxia severity using ICARS moderately correlates with NAA/Cr in the middle cerebellar peduncle (*r* = −0.45) and strongly correlates with the dentate nucleus (*r* = −0.50) (Peng et al., 2019). The discrepancy in findings using different clinical measures provides an avenue for future study on the reliability of SARA and ICARS in correlating with the NAA/Cr ratio.

Ataxia severity using SARA showed no correlation between SARA and Cho/Cr ratio in the total cerebellum (Wang et al., 2012; Adanyeguh et al., 2015). Regarding specific cerebellar regions, SARA moderately correlates with Cho/Cr ratio in the cerebellar hemispheres (*r* = −0.39) and dentate nucleus (*r* = 0.36), and strongly correlates with the vermis (*r* = 0.93) (Lei et al., 2011). Also, ataxia severity using ICARS moderately correlates with Cho/Cr ratio in the dentate nucleus (*r* = −0.37) (Peng et al., 2019). The correlation between Cho/Cr ratio in the dentate nucleus and ataxia severity measured by SARA and ICARS is similar. The finding suggests that the difference in the predictive value between SARA and ICARS for Cho/Cr ratio was minimal. Therefore, a strong correlation between Cho/Cr ratio in the vermis and ataxia severity is unlikely attributed to the type of scale, thereby representing a reliable biomarker.

Lastly, a study reported that ataxia severity using SARA moderately correlates with the NAA/Cho ratio cerebellar hemispheres (*r* = 0.46) and vermis (*r* = 0.37) (Lei et al., 2011).

###### Summary.

Correlation between ataxia severity and MRS changes was scarcely studied in SCA3 patients. Specifically, only a single study established the correlation between ataxia severity and individual metabolite change in the brainstem of SCA3 patients (Adanyeguh et al., 2015). At present, changes in the metabolite ratios are not uniform across the cerebellum. Specifically, reduced Cho/Cr in the vermis may represent a more accurate MRS biomarker for ataxia severity in SCA3 patients due to its strong correlation with SARA scores.

##### DTI

###### Cerebrum (Subcortical).

A study reported that ataxia severity using ICARS moderately correlates with FA of the posterior thalamic radiation (*r* = 0.44) (Wu et al., 2017).

###### Brainstem.

A study reported that ataxia severity using SARA moderately correlates with FA of the left cortical-spinal tract (*r* = −0.47), and strongly correlates with FA of the right cortical-spinal tract (*r* = −0.51) (Meira et al., 2020). Another study reported that ataxia severity using ICARS strongly correlates with the FA of the medial lemniscus (*r* = −0.52) (Wu et al., 2017).

###### Cerebellum.

A study reported no correlation between ataxia severity using SARA and FA in the total cerebellum (Guimarães et al., 2013). Ataxia severity using ICARS moderately to strongly correlates with FA in the superior (*r* = −0.43 ~ −0.64) (Wu et al., 2017; Peng et al., 2019), middle (*r* = −0.42), and inferior (*r* = −0.60) cerebellar peduncles (Peng et al., 2019).

###### Summary.

There is no established correlation between SARA and FA in the total cerebellum, whereas ataxia severity using ICARS correlated with FA in several white matter tracts. These findings suggest that FA in white matter tracts indicates ataxia severity than FA of other brain regions, including the total cerebellum. These findings remain preliminary, and each tract's contribution deserves further exploration.

#### Motor (Non-ataxia)

There was no association between Burke–Marsden–Fahn's Dystonia Rating Scale (BMFDRS) and MRI techniques, including structural MRI and DTI. Similar to ataxia scales, one possible explanation for the lack of correlation is that the BMFDRS is susceptible to other movement difficulties (Kuiper et al., 2016). Nevertheless, the study reported the presence of precentral and paracentral cortices atrophy and more severe thalamus atrophy in SCA3 patients with dystonia compared to their non-dystonic counterparts (Nunes et al., 2015). In addition, midbrain atrophy may also contribute to dystonia in SCA3 (Vidailhet et al., 1999). Further studies are needed to identify brain regions that contribute to dystonia in SCA3 patients.

#### Neurocognition

We conducted an a-priori categorization of neuropsychological tests into three neurocognitive domains informed by theory and convention (Strauss et al., 2006; Lezak et al., 2012). These domains were (1) general intelligence, (2) working memory (WM), and (3) executive aspect of language. All of which were related to the executive function (EF) (Miyake et al., 2000; Van Aken et al., 2016; Tamura et al., 2018).

#### General Intelligence

General intelligence is a construct that represents a person's verbal and non-verbal reasoning abilities (Kamphaus, 2005).

##### Structural MRI

###### Cerebrum (Cortical).

One study reported that verbal reasoning using the Similarities subtest strongly correlates with the cortical thickness of the left precentral gyrus (*r* = 0.82) and right superior occipital gyrus (*r* = 0.84) (De Rezende et al., 2015). On the other hand, non-verbal reasoning using Raven's Progressive Matrices (RPM) strongly correlates with the cortical thickness of the left middle occipital gyrus (*r* = 0.90) (De Rezende et al., 2015). Contradictorily, these measures of general intelligence are commonly associated with frontal and parietal cortices but not precentral and occipital gyri (Gläscher et al., 2009). Therefore, rather than playing a direct role in general intelligence, cortical thickness in the precentral and occipital gyri may share a parallel neurodegenerative process in the SCA3 neuropathology that impedes general intelligence.

##### MRS

###### Cerebellum.

One study reported that the RPM moderately correlates with Glu (*r* = −0.42) and Glx (*r* = −0.41) in the cerebellum (Lopes et al., 2013). The correlation between the cerebellar metabolites and RPM may be attributed to the extensive reciprocal connections between the cerebellum and areas of the cerebrum (Bostan et al., 2013), which disrupts cognitive function (Stoodley and Schmahmann, 2010). Negative correlations between metabolites and RPM are likely related to the processing techniques such as global signal regression than actual anti-correlated activity (Murphy et al., 2009).

#### Working Memory

##### MRS

###### Cerebellum.

One study reported that auditory WM using Digit Span (DS) moderately correlates with the NAA (*r* = 0.42) and NAA + NAAG (*r* = 0.43) in the cerebellum (Lopes et al., 2013), consistent with the finding that the cerebellum plays a role in the processing of auditory WM (Tomlinson et al., 2014).

##### DTI

###### Brainstem.

One study reported that auditory WM using DS moderately correlates with the FA of the brainstem (*r* = 0.49) (Lopes et al., 2013). The relationship between the brainstem and WM is shown when WM plays an essential role in the early precortical sensory processing, in this case, auditory-evoked brainstem response (Sörqvist et al., 2012). The finding suggests that brainstem degeneration is associated with the WM impairment in SCA3.

#### Language

##### MRS

###### Cerebellum.

One study reported that semantic verbal fluency strongly correlates with the PCh (*r* = 0.66) and GPC + PCh (*r* = 0.69) in the cerebellum (Lopes et al., 2013). This finding highlights the cerebellum's role in motor and sequential speech processing (Leggio et al., 2000). Specifically, an impaired cerebellar cortex-ventral dentate nucleus-thalamus-prefrontal cortex circuit in SCA3 is heavily implicated in strategic word retrieval (Tamura et al., 2018).

#### Summary

Existing findings on the correlations between neurocognition and neurodegenerative process in SCA3 are restricted to EF. The correlations between neurocognition the cerebellum in SCA3 were at least moderate. This finding is consistent with the cerebellum's role in EF processing due to its extensive reciprocal connections with areas of the cerebrum, including the prefrontal and posterior parietal cortex and the basal ganglia (Stoodley and Schmahmann, 2010; Bostan et al., 2013). Contradictorily, no correlation was found with the prefrontal-striatal circuit, which is impaired in SCA3 and plays a crucial role in higher-order cognitive functions (Alexander et al., 1986; Yap et al., 2022b). Instead, strong correlations were found between general intelligence and precentral gyrus and superior occipital gyrus (De Rezende et al., 2015). This finding determines whether these regions are simultaneously affected alongside the distributed network involved in the EF processing in SCA3.

## Discussion

In this review, we demonstrated widespread brain degeneration with moderate correlations between MRI features in several regions and clinical findings in SCA3 patients. Regardless of the MRI techniques, most studies focused on the brainstem and cerebellum. This trend aligns with the body of evidence that suggests that these regions are affected earlier than the cerebrum (Rezende et al., 2018). These early degenerations are consistent with ataxia being the key symptom observed in SCA3 (Bodranghien et al., 2016). Interestingly, the structural loss of the cerebellum in SCA3 was less affected than other SCA subtypes (Schulz et al., 2010). Instead, studies reported degenerative atrophy of various cerebral structures in SCA3, including cortical and sub-cortical regions (Schulz et al., 2010; D'Abreu et al., 2012; De Rezende et al., 2015). As the disease progresses, the SCA3 neuropathology gradually affects the cerebrum, in which extensive atrophy is observed in the cerebral cortex and basal ganglia in the late stages of SCA3 (Rezende et al., 2018). This process results in widespread brain degeneration, contributing to and exacerbating non-ataxia and cognitive impairment in SCA3 (Lindsay and Storey, 2017).

We also examined the MRI abnormalities that may explain the clinical manifestations in SCA3 based on correlation studies. SARA and ICARS represent the most frequently used ataxia scales for SCA3 patients. Regardless of the MRI techniques, the correlation findings were similar between SARA and ICARS (Rüb et al., 2003; Eichler et al., 2011; Bodranghien et al., 2016). Consistently, SARA and ICARS are equally reliable in measuring ataxia severity in SCA3. However, a relatively higher inter-rater reliability, functional relevance, and timesaving nature confer advantages to the SARA (Zhou et al., 2011).

In contrast to the ataxia symptoms, the MRI correlates to the cognitive impairment in SCA3 have not been comprehensively evaluated. Specifically, existing findings focused only on the EF of SCA3 patients. Interestingly, some MRI abnormalities showed associations between EF and the left precentral gyrus and right superior occipital gyrus (De Rezende et al., 2015), rather than the frontal and parietal cortices (Gläscher et al., 2009). We postulate that these regions shared a parallel neurodegenerative process with the cognitive decline in SCA3. Alternatively, these regions may play a pivotal role in the distributed networks serving these neurocognitive domains.

At present, the correlations between the structural MRI, MRS, and DTI changes in several regions and ataxia and cognitive impairment were mainly in the moderate range (*r* = 0.30–0.49) (Cohen, 2013). In support, a study that used 3D-FD analysis showed a similar correlation between the cerebellar cortex and SARA scores (*r* = −0.33) (Wang et al., 2020). In addition, there was no correlation between dystonia using BMFDRS and MRI techniques in SCA3 patients. The presence of clinical syndrome may compound each other; that is, ataxia and dystonia may exacerbate each other when measured with their respective clinical scales. Likewise, motor impairment is often a confounding factor for clinical scales from truly reflecting cognitive impairment (Yap et al., 2022b). This issue is often exacerbated as few tests assess only a single domain (i.e., true process-pure tests), thereby affecting the test utility in the cerebellar patient population. Nevertheless, these symptoms are part of the clinical syndrome of SCA3 and are nearly impossible to disentangle from studying one another.

In addition, the involvement of multiple brain regions in SCA3 may also complicate the correlational findings. For example, several different brain regions may contribute to ataxia severity, including the thalamus, pons, and cerebellum (Rüb et al., 2003; Eichler et al., 2011; Bodranghien et al., 2016). This finding may explain moderate correlations between each brain region and the corresponding clinical findings. More importantly, it may represent the difference between isolated cerebellar (e.g., cerebellar stroke) and complex cerebrocerebellar diseases (e.g., SCA3), to which the latter involves both cerebellar and cerebral degeneration.

We postulate that in a complex cerebrocerebellar disease like SCA3, degeneration in any part of the brain region and its connections in the same distributed network may result in the same clinical symptom (Benowitz and Carmichael, 2010), despite each playing a different role in the process. For example, severe ataxia is not only observed when the cerebellum is impaired (Bodranghien et al., 2016); the disruption in the connection between the cerebellum and motor cortex in SCA3 also results in the same symptom (Maas et al., 2021). Specifically, the cerebellum projects to the contralateral premotor and primary motor cortices with sensory and motor information necessary to adapt movements in response to feedback, termed the motor network (Spampinato et al., 2020). Likewise, disruption in the cerebellar-cerebral network and prefrontal-striatal circuit were involved in the cognitive impairment in SCA3 (Lindsay and Storey, 2017). In this case, the cerebellum serves as a hub in the network that prepares for neural processing to optimize the action sequencing on the EF task (Beuriat et al., 2020). Cerebellar degeneration compounds the EF impairment due to the frontal lobe atrophy in SCA3 patients (Klinke et al., 2010).

## Implications

This review highlights two research gaps in the literature. First, the brain-behavior relationship in SCA3 has not been comprehensively evaluated using MRI-based brain networks. We postulate that a distributed network, rather than an individual brain region such as the cerebellum, is responsible for the clinical syndrome in SCA3 (Beuriat et al., 2020; Spampinato et al., 2020). In support, the 3D-FD analysis showed that SCA3 neuropathology involves dissociation in the cerebellar-cerebral network (Huang et al., 2017; Jao et al., 2019b). Second, most studies mainly aimed at investigating the structural loss in SCA3 patients, including attempts to increase the sensitivity of the scan, such as texture analysis (De Oliveira et al., 2010) and neuromelanin contrast (Nakata et al., 2020), whereas functional activation has been scarcely studied. Functional activation allows the inference of brain regions and networks involved in a particular motor or cognitive process (Berman et al., 2006). At present, a relaxometry study showed decreased T2-relaxation values in the white matter of the right cerebellar hemisphere alongside structural loss (Guimarães et al., 2013), and a task-based fMRI study showed that a paced motor task elicited increased activations in the motor network SCA3 patients irrespective of gray matter loss (Duarte et al., 2016). These findings are similar to other neurodegenerative diseases such as Alzheimer's disease, which increases activation in early disease stage (Yap et al., 2017). Together with the presence of brain structural and metabolic changes before the clinical manifestation (Wu et al., 2017; Joers et al., 2018; Rezende et al., 2018), the findings imply functional reorganization of the brain and that compensatory activation is in play (Duarte et al., 2016), which gradually decline as the disease progresses (Yap et al., 2017). Notably, the compensatory activation may also obscure the correlations between brain structural and metabolic changes and clinical findings, which may also explain the moderate association strengths. Therefore, we need more findings on the fMRI for SCA3 patients, especially the resting-state networks.

The importance of these findings points to the pivotal role of adaptive or maladaptive compensatory activation. A maladaptive compensatory activation is shown when dysfunction in the brain region results in its inability to downregulate other brain regions in the same distributed network. Therefore, the increased activation disrupts clinical performance (Franzen et al., 2013; Jones et al., 2016). Conversely, adaptive compensatory activation may imply that neuroplasticity is present in SCA3 patients, providing support for early physical and cognitive rehabilitation. While restorative neurorehabilitation has been widely studied in the SCA3 population, findings for non-pharmacological approaches for SCA, such as cognitive rehabilitation and psychotherapy on other aspects of psychological symptoms, are lacking (Yap et al., 2022a).

## Limitations

This review has several limitations. Firstly, different MRI techniques have not been consistently studied across different brain regions in the SCA3 population, specifically the cerebrum. Likewise, correlations between clinical impairment and brain regions were not consistently reported. Therefore, we could not conclude whether the absence of correlations was due to non-significant findings or was not assessed. Secondly, SCA3 patients are commonly characterized by peripheral nerve involvement, which further exacerbating test performances through non-ataxia symptoms such as muscle weakness and sensory disturbances (Van De Warrenburg et al., 2004). It is essential to rule out these factors before correlating the brain MRI abnormalities and clinical syndrome. Thirdly, a potential problem with MRI studies involved multiple comparisons, which increases the likelihood of false positives (Lindquist and Mejia, 2015). This problem applies to comparing brain regions between SCA3 and HC and the number of correlations conducted. Therefore, Bonferroni correction may help address the problem (Lindquist and Mejia, 2015). However, it is also possible that none of the true positives would survive multiple comparison corrections. Lastly, there is a lack of knowledge on the brain degenerative patterns between SCA3 patients from Western and East Asian countries. The difference in the brain structure may affect the degenerative pattern, although this has not been studied (Tang et al., 2018). In addition, factors such as test norms, age, gender, culture, education level, and functional level may further affect the degenerative pattern and correlations between MRI and clinical findings.

## Conclusion

SCA3 neuropathology contributes to widespread brain degeneration. We speculate that the connectivity between different brain regions in distributed networks is responsible for motor and neurocognitive function in SCA3. However, this topic remains scarcely studied in SCA3. Therefore, future studies should investigate possible functional connectivity abnormalities in SCA3 using fMRI.

## Data Availability Statement

The original contributions presented in the study are included in the article/Supplementary Material, further inquiries can be directed to the corresponding author/s.

## Author Contributions

Funding acquisition was performed by NM. Literature search was performed by KY and HA. The first draft of this review was written by KY. All authors critically revised the work, contributed to the study conception and design, and read and approved the final version of the review.

## Funding

This work was supported by the Dana Impak Perdana Grant (DIP-2019-007) received by NM from Universiti Kebangsaan Malaysia.

## Conflict of Interest

The authors declare that the research was conducted in the absence of any commercial or financial relationships that could be construed as a potential conflict of interest.

## Publisher's Note

All claims expressed in this article are solely those of the authors and do not necessarily represent those of their affiliated organizations, or those of the publisher, the editors and the reviewers. Any product that may be evaluated in this article, or claim that may be made by its manufacturer, is not guaranteed or endorsed by the publisher.
