# Supplementary material for: Magnetic Resonance Imaging and Its Clinical Correlation in Spinocerebellar Ataxia Type 3: A Systematic Review
Source: Front Neurosci. 2022 Jun 10;16:859651. doi: 10.3389/fnins.2022.859651 (PMC9226753; doi:10.3389/fnins.2022.859651)
Supplement: Supplementary file 1 [file Table_1.docx]

Supplementary Table 1

*Risk of bias assessment on included studies adapted from The National Institutes of Health (NIH) quality assessment tool of case-control studies.*

| First author, year | #1 | #2 | #3 | #4 | #5 | #6 | #7 | #8 | #9 | #10 | #11 | #12 |
| --- | --- | --- | --- | --- | --- | --- | --- | --- | --- | --- | --- | --- |
| (Bürk et al., 1996) | 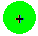 | 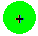 | 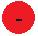 | 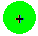 | 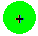 | 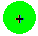 | 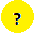 | 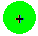 | 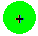 | 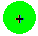 | 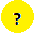 | 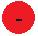 |
| (Etchebehere et al., 2001) | 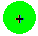 | 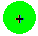 | 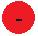 | 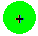 | 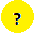 | 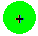 | 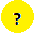 | 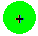 | 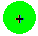 | 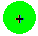 | 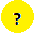 | 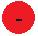 |
| (Yoshizawa et al., 2003) | 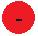 | 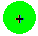 | 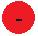 | 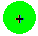 | 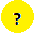 | 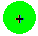 | 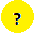 | 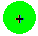 | 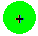 | 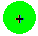 | 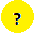 | 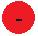 |
| (Liang et al., 2009) | 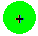 | 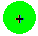 | 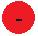 | 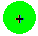 | 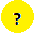 | 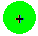 | 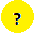 | 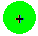 | 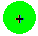 | 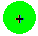 | 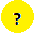 | 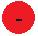 |
| (Schulz et al., 2010) | 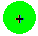 | 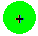 | 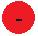 | 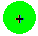 | 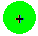 | 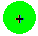 | 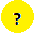 | 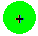 | 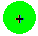 | 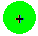 | 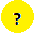 | 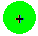 |
| (Camargos et al., 2011) | 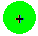 | 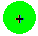 | 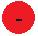 | 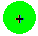 | 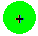 | 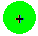 | 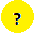 | 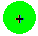 | 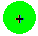 | 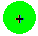 | 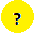 | 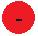 |
| (D’Abreu et al., 2011) | 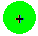 | 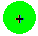 | 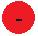 | 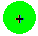 | 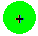 | 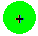 | 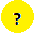 | 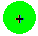 | 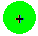 | 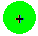 | 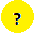 | 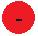 |
| (Lei et al., 2011) | 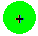 | 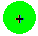 | 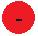 | 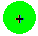 | 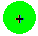 | 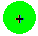 | 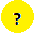 | 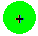 | 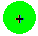 | 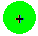 | 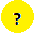 | 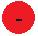 |
| (D’Abreu et al., 2012) | 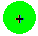 | 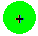 | 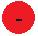 | 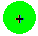 |  |  |  |  |  |  |  |  |
| (Lirng et al., 2012) |  |  |  |  |  |  |  |  |  |  |  |  |
| (Ogawa et al., 2012) |  |  |  |  |  |  |  |  |  |  |  |  |
| (Wang et al., 2012) |  |  |  |  |  |  |  |  |  |  |  |  |
| (Guimarães et al., 2013) |  |  |  |  |  |  |  |  |  |  |  |  |
| (Lopes et al., 2013) |  |  |  |  |  |  |  |  |  |  |  |  |
| (Chen et al., 2014) |  |  |  |  |  |  |  |  |  |  |  |  |
| (Kang et al., 2014) |  |  |  |  |  |  |  |  |  |  |  |  |
| (Adanyeguh et al., 2015) |  |  |  |  |  |  |  |  |  |  |  |  |
| (de Rezende et al., 2015) |  |  |  |  |  |  |  |  |  |  |  |  |
| (Nunes et al., 2015) |  |  |  |  |  |  |  |  |  |  |  |  |
| (Hernandez-Castillo et al., 2017) |  |  |  |  |  |  |  |  |  |  |  |  |
| (Wu et al., 2017) |  |  |  |  |  |  |  |  |  |  |  |  |
| (Rezende et al., 2018) |  |  |  |  |  |  |  |  |  |  |  |  |
| (Jao et al., 2019a) |  |  |  |  |  |  |  |  |  |  |  |  |
| (Jao et al., 2019b) |  |  |  |  |  |  |  |  |  |  |  |  |
| (Peng et al., 2019) |  |  |  |  |  |  |  |  |  |  |  |  |
| (Arruda et al., 2020) |  |  |  |  |  |  |  |  |  |  |  |  |
| (Guo et al., 2020) |  |  |  |  |  |  |  |  |  |  |  |  |
| (Meira et al., 2020) |  |  |  |  |  |  |  |  |  |  |  |  |
| (Inada et al., 2021) |  |  |  |  |  |  |  |  |  |  |  |  |

_Green circle: low risk; yellow circle: unclear: cannot determine, not applicable, or not reported; red circle: high risk._

^Criterion 1: Appropriate research question.^

^Criterion 2: Define study population.^

^Criterion 3: Sample size justification.^

^Criterion 4: Groups recruited from the same population.^

^Criterion 5: Inclusion and exclusion criteria prespecified and applied uniformly.^

^Criterion 6: Case and control definitions.^

^Criterion 7: Random selection of study participants.^

^Criterion 8: Concurrent controls.^

^Criterion 9: Exposure assessed prior to outcome measurement.^

^Criterion 10: Define exposure measures and assessment.^

^Criterion 11: Blinding of exposure assessors.^

^Criterion 12: Statistical analysis for confounding variables.^

Supplementary Table 2

*Characteristics of included studies of SCA 3 in this review.*

| First author, year, country | Study design | Sample size | Main clinical characteristics | MRI Tesla | Clinical measure(s) [SCA3] | MRI measure(s) [SCA3] | MRI outcome(s) [SCA3 vs. HC] | Clinical vs. MRI correlation(s) [SCA3]  Effect size(s) [Pearson’s *r*] |
| --- | --- | --- | --- | --- | --- | --- | --- | --- |
| (Bürk et al., 1996)  Germany | Case control  Level: III-2 | SCA1: 9  SCA2: 8  SCA3: 12  HC: 36 | Mean age (years):  HC: 46.1 ± 2.4  Mean onset age (years):  SCA1: 34.0 ± 4.3  SCA2: 34.6 ± 4.6  SCA3: 41.3 ± 3.7  Disease duration (years):  SCA1: 10.3 ± 1.5  SCA2: 8.9 ± 1.6  SCA3: 7.0 ± 1.3  Gender (M/F): -  CAG repeat length: -  Education: - | 1.5 | - | Structural MRI  Volumetric | Brainstem  Atrophy in pontine base, medulla oblongata.  Cerebellum  Atrophy in cerebellar vermis and middle cerebellar peduncle.  Spinal cord  Atrophy in cervical spinal cord.  Ventricle  Enlargement of fourth ventricle. | - |
| (Etchebehere et al., 2001)  Brazil | Case control  Level: III-2 | SCA3: 12  HC: 17 | Mean age (years):  SCA3: 39  HC: 32.2  Mean onset age (years): -  Disease duration (years):  SCA3: 8.7 ± 5.3  Gender (M/F):  SCA3: 8/4  HC: 4/13  CAG repeat length:  SCA3: 72.3 ± 3.5  Education: - | 2.0 | - | Structure MRI  Volumetric | Cerebellum  Atrophy in vermis and cerebellar hemispheres. | - |
| (Yoshizawa et al., 2003)  Japan | Case control  Level: III-2 | SCA3: 13  HC: 17 | Mean age (years):  SCA3: 45.5 ± 3.3  HC: 49.2 ± 3.8  Mean onset age (years): -  Disease duration (years):  SCA3: 11.9 ± 6.5  Gender (M/F):  SCA3: 8/4  HC: 6/11  CAG repeat length:  SCA3: 77.0 ± 7.0  Education: - | 1.5 | - | Structure MRI  Volumetric | Brainstem  Atrophy in pontine tegmentum (greater) and pontine base. | - |
| (Liang et al., 2009)  China | Case control  Level: III-2 | SCA3: 32  HC: 36 | Mean age (years):  SCA3: 39.9 ± 7.4 HC: 36.5 ± 10.2  Mean onset age (years):  SCA3: 34.9 ± 6.6  Disease duration (years):  SCA3: 4.8 ± 3.6  Gender (M/F):  SCA3: 16/16  HC: 16/20  CAG repeat length:  SCA3: 71.8 ± 2.6  Education: - | 1.5 | ICARS  SARA | Structure MRI   - Volumetric | Brainstem  Atrophy in the whole brainstem except antero-posterior pons and medulla.  Reduced pons to prepontine cistern and medulla to anterior medullary cistern ratios.  No change in the antero-posterior midbrain to diameter between midbrain and dorsum sellae ratio.  Cerebellum  Atrophy in the cerebellum except antero-posterior and supero-inferior regions.  Ventricle  Enlargement of the supero-inferior region of the fourth ventricle. | Correlation between ICARS and:  Brainstem  Antero-posterior midbrain (*r* = - .53)  Antero-posterior pons (*r* = -.57)  Antero-posterior midbrain to midbrain and dorsum sellae ratio (*r* = -.42)  Correlation between SARA and:  Brainstem  Antero-posterior midbrain (*r* = - .44)  Antero-posterior pons (*r* = -.55)  Cerebellum  Supero-inferior regions of cerebellum (*r* = -.42) |
| (Schulz et al., 2010)  Germany | Case control  Level: III-2 | SCA1: 48  SCA3: 24  SCA6: 10  HC: 31 | Mean age (years):  SCA1: 44.3 ± 11.9  SCA3: 47.3 ± 11.4  SCA6: 61.9 ± 10.2  HC: 49.8 ± 16.2  Mean onset age (years): -  Disease duration (years):  SCA1: 8.1 ± 4.5  SCA3: 11.7 ± 6.0  SCA6: 8.8 ± 4.9  Gender (M/F):  SCA1: 31/17  SCA3: 11/13  SCA6: 9/1  HC: 14/17  CAG repeat length: -  Education: - | 1.5 | SARA  UHDRS | Structure MRI   - VBM - Volumetric | *VBM*  Cerebrum  No atrophy in the GM of cerebral hemispheres (cortical).  No atrophy in the GM of pons and basal ganglia (subcortical).  Brainstem  Atrophy in the WM of brainstem, midbrain, and pons.  Cerebellum  Atrophy in the GM cerebellar hemispheres and vermis (except lobules VII and VIII).  Atrophy in the white matter of cerebellar peduncles and hemispheres (lobules I-VI).  No atrophy in the white matter of inferior hemispheric lobules.  *Volumetric*  Cerebrum  Atrophy in the putamen and caudate nucleus (subcortical).  No atrophy in the cerebral hemispheres except temporal lobe.  Brainstem  Atrophy in brainstem, including midbrain, pons and medulla.  Cerebellum  Atrophy in the cerebellar hemisphere and vermis. | Correlation between SARA and:  Cerebrum (subcortical)  Putamen + caudate nucleus (*r* = -.36)  Caudate nucleus (*r* = -.46)  Brainstem  Total brainstem (*r* = -.68)  Midbrain (*r* = -.47)  Pons (*r* = -.56)  Medulla (*r* = -.48)  Cerebellum  Total cerebellum (*r* = -.45)  Cerebellar hemispheres (*r* = -.46)  Correlation between UHDRS and:  Brainstem  Total brainstem (*r* = -.58)  Midbrain (*r* = -.46)  Pons (*r* = -.49)  Cerebellum  Total cerebellum (*r* = -.46)  Cerebellar hemispheres (*r* = -.48) |
| (D’Abreu et al., 2011)  Brazil | Case control  Level: III-2 | SCA3: 45  HC: 52 | Mean age (years):  SCA3: 46.2 ± 13.2  Mean onset age (years):  SCA3: 36.2 ± 12.1  Disease duration (years):  SCA3: 9.9 ± 6.1  Gender (M/F):  SCA3: -  CAG repeat length:  SCA3: 66.0  Education: - | 2.0 | - | Structure MRI   - Volumetric | Cerebrum  Atrophy in the thalamus (subcortical). | - |
| (Lei et al., 2011)  China | Case control  Level: III-2 | SCA3: 36  HC: 27 | Mean age (years):  SCA3: 36.7 ± 6.0  HC: 31.0 ± 7.4  Mean onset age (years):  SCA3: 31.4 ± 6.3  Disease duration (years):  SCA3: 4.5 ± 2.6  Gender (M/F):  SCA3: 18/18  HC: 13/14  CAG repeat length:  SCA3: 75.5 ± 4.3  Education: - | 1.5 | SARA | MRS | Cerebellum  Reduced NAA/Cr in the cerebellar cortex, dentatum, vermis, and medipeduncle.  Reduced NAA/Cho in the dentatum and vermis.  *No change in Cho/Cr | Correlation between SARA and:  Cerebellum  Dentate nucleus   - NAA/Cr (*r* = .45) - Cho/Cr (*r* = .36)   Cortex   - Cho/Cr (*r* = -.39) - NAA/Cho (*r* = .46)   Vermis   - Cho/Cr (*r* = .93) - NAA/Cho (*r* = .37)   Medipeduncle   - NAA/Cr (r = .95) |
| (Camargos et al., 2011)  Brazil | Case control  Level: III-2 | SCA3: 15  HC: 15 | Mean age (years):  SCA3: 44.3 ± 15.6  Mean onset age (years): -  Disease duration (years):  SCA3: 8.7 ± 5.8  Gender (M/F):  SCA3: 8/7  HC: 8/7  CAG repeat length:  SCA3: 72.7 ± 5.3  Education: - | 1.5 | ICARS | Structure MRI   - Volumetric | Brainstem  Atrophy in total brainstem, midbrain, pons, and medulla.  Cerebellum  Atrophy in total cerebellum, cerebellar hemispheres, and vermis.  No atrophy in cerebellar tonsil. | Correlation between ICARS and:  Brainstem  Total brainstem (*r* = -.62)  Pons (*r* = -.68)  Cerebellum  Total cerebellum (*r* = -.60)  Right hemisphere (*r* = -.49)  Left hemisphere (*r* = -.63)  Vermis region 2 (*r* = -.52) |
| (D’Abreu et al., 2012)  Brazil | Case control  Level: III-2 | SCA3: 45  HC: 51 | Mean age (years):  SCA3: 47.0 ± 12.2  HC: 44.1 ± 11.8  Mean onset age (years):  SCA3: 37.0 ± 11.1  Disease duration (years):  SCA3: 10.0 ± 6.1  Gender (M/F):  SCA3: 30/15  CAG repeat length:  SCA3: 72  Education: - | 2.0 | ICARS | Structure MRI   - VBM | Cerebrum  Atrophy in the GM of lentiform nucleus, caudate nucleus, claustrum, and limbic lobe (cingulate cortex, parahippocampal gyrus), and thalami (subcortical).  Atrophy in the GM of frontal lobes (precentral; inferior, superior and middle frontal gyrus), parietal lobes (postcentral gyrus; precuneus; inferior and superior angular, and supramarginal gyrus), temporal lobes (fusiform gyrus; insula; middle, superior temporal gyrus), and occipital gyrus (cuneus, inferior occipital gyrus, lingual gyrus, middle occipital gyrus, superior occipital gyrus) (cortical).  Brainstem  Atrophy in the GM of brainstem (medulla, pons, and midbrain).  Cerebellum  Atrophy in the GM of cerebellum and vermis. | Correlation between ICARS and:  Cerebrum  Frontal lobe (*r* = .62)  Parietal lobe (*r* = .57)  Temporal lobe (*r* = .72)  Occipital lobe (*r* = .44)  Cerebellum  Cerebellum and vermis (*r* = .73) |
| (Lirng et al., 2012)  Taiwan | Case control  Level: III-2 | SCA1: 4  SCA2: 16  SCA3: 58  SCA6: 10  SCA17: 6  MSA-C: 62  HC: 44 | Mean age (years):  SCA1: 56.0 ± 2.3  SCA2: 44.9 ± 16.9  SCA3: 50.0 ± 12.7  SCA6: 56.7 ± 11.3  SCA17: 52.0 ± 15.5  MSA-C: 62.6 ± 7.1  HC: 51.1 ± 17.9  Mean onset age (years): -  Disease duration (years):  SCA1: 12.3 ± 12.9  SCA2: 6.5 ± 4.0  SCA3: 8.9 ± 6.2  SCA6: 8.3 ± 7.7  SCA17: 3.0 ± 3.2  MSA-C: 5.3 ± 3.6  Gender (M/F): -  CAG repeat length:  SCA1: 43.8 ± 4.4  SCA2: 42.9 ± 6.2  SCA3: 73.2 ± 3.9  SCA6: 23.6 ± 1.1  SCA17: 44.5 ± 2.2  Education: - | 1.5 | SARA | MRS | Cerebellum  Reduced NAA/Cr and NAA/Cho in the cerebellar hemispheres and vermis.  *No change in Cho/Cr | *Did not distinguish between SCA3 from other SCA subtypes. |
| (Ogawa et al., 2012)  Japan | Case control  Level: III-2 | SCA3: 17  DRPLA: 8  SCA6: 7  HC: 17 | Mean age (years):  SCA3: 52.4 ±  12.7  DRPLA: 48.3 ± 19.1  SCA6: 58.0 ± 9.6  HC: 56.0 ± 7.2  Mean onset age (years): -  Disease duration (years):  SCA3: 11.1 ± 6.0  DRPLA: 8.5 ± 4.2  SCA6: 11.5 ± 9.8  Gender (M/F):  SCA3: 8/9  DRPLA: 4/4  SCA6: 2/5  HC: 8/9  CAG repeat length:  SCA3: 68.2 ± 5.6  DRPLA: 57.8 ± 3.5  SCA6: 23.5 ± 1.6  Education: - | 1.5 | ICARS | Structure MRI   - Volumetric | Brainstem  Atrophy in the facial colliculus and pontine tegmentum.  Cerebellum  Atrophy in the superior cerebellar peduncle. | - |
| (Wang et al., 2012)  Taiwan | Case control  Level: III-2 | SCA2: 12  SCA3: 43  SCA6: 8  HC: 44 | Mean age (years):  SCA2: 50.8 ± 15.2  SCA3: 48.8 ±  11.4  SCA6: 56.3 ± 9.8  HC: 51.1 ± 18.0  Mean onset age (years):  SCA2: 44.3 ± 17.3  SCA3: 40.1 ± 10.5  SCA6: 47.9 ± 8.4  Disease duration (years):  SCA2: 6.5 ± 4.0  SCA3: 8.7 ± 6.2  SCA6: 8.3 ± 7.7  Gender (M/F): -  CAG repeat length:  SCA2: 40.2 ± 3.5  SCA3: 73.1 ± 4.0  SCA6: 23.5 ± 1.0  Education: - | 1.5 | SARA | MRS | Cerebellum  Reduced NAA/Cr in right and left cerebellar hemispheres and vermis.  *No change in Cho/Cr. | No correlation between NAA/Cr and Cho/Cr with SARA scores. |
| (Guimarães et al., 2013)  Brazil | Case control  Level: III-2 | SCA3: 38  HC: 38 | Mean age (years):  SCA3: 52.8 ±  12.7  HC: 48.9 ±  12.1  Mean onset age (years):  SCA3: 40.4 ± 12.1  Disease duration (years):  SCA3: 9.3 ± 2.7  Gender (M/F):  SCA3: 21/17  HC: 20/18  CAG repeat length:  SCA3: 68.1 ± 4.5  Education: - | 3.0 | SARA  ICARS | Structure MRI   - VBM   DTI | **Structure MRI**  *VBM*  Brainstem  Atrophy in the GM of pons, pyramids, and medulla.  Atrophy in the WM of right brainstem, medulla, pyramids, and pons.  Cerebellum  Atrophy in the GM of posterior cerebellum, vermis, tonsil, inferior semilunar lobule, declive, uvula, fastigium, and tuber.  Atrophy in the WM of tonsil, posterior lobe, culmen, declive, vermis, dentate, uvula, posterior cingulate, fastigium, tuber, nodule, and cerebellar peduncles.  **DTI**  Cerebrum  Increased RD in the thalamus, bilateral cerebral white matter (subcortical), and frontal and temporal lobes (cortical).  Increased AD in the right thalamus (subcortical).  Brainstem  Reduced FA in the pons and midbrain.  Increased RD in the pons and midbrain.  Increased AD in the brainstem and midbrain.  Cerebellum  Reduced FA in the bilateral anterior lobe, right posterior lobe, nodule, culmen, dentate, fastigial, lingual, and superior, middle, and inferior cerebellar peduncles.  Increased RD in the bilateral anterior lobe, posterior lobe, culmen, fastigium, dentate, tonsil, lingual, pyramids, uvula, declive, and cerebellar peduncles.  Increased AD in the bilateral cerebellum (anterior and posterior lobes), culmen, fastigium and lingual.  *Decrement more pronounced in brainstem than cerebellum. | *Negative correlations between SARA and:  *VBM*  Brainstem  Midbrain GM  Pons GM  Bilateral brainstem WM  Midbrain WM  Cerebellum  Anterior & posterior GM  Anterior & posterior WM  Culmen WM  Vermis WM  **DTI**  *No correlation reported  Correlation between ICARS and:  *VBM*  Brainstem  Bilateral brainstem WM  Midbrain WM  Cerebellum  Anterior & posterior WM  Culmen WM  Vermis WM |
| (Lopes et al., 2013)  Brazil | Case control  Level: III-2 | SCA3: 32  HC: 32 | Mean age:  SCA3: 46.8 ± 11.5  HC: 47.0 ± 12.1  Mean onset age:  SCA3: 36.7 ± 10.9  Disease duration (years):  SCA3: 10.1 ± 5.8  Gender (M/F):  SCA3: 18/20  HC: 14/17  CAG repeat length:  SCA3: 69.0 ± 5.0  Education:  SCA3: 10.2 ± 3.8  HC: 10.4 ± 4.3 | 3.0 | RAVLT  RPM  CBTT  DS  Similarities  PC  Verbal Fluency (Semantic; Animal)  LM  VPA  Figural Memory  Pseudo Repetition Word Test  BNT  WCST  BDI | Structure MRI   - VBM   MRS (Cerebellum)  DTI | **Structure MRI**  *VBM*  Cerebrum  Reduced GM of the left posterior cingulum and right putamen (subcortical); and left superior parietal lobe and left precentral gyrus (cortical).  Cerebellum  Reduced GM of right and left cerebellum  **MRS**  Cerebellum  Reduced NAA/Cr + PCr, NAA + NAAG/Cr + PCr, and Glx/Cr + PCr in the cerebellum.  *No reduction in Glu/Cr + PCr, PCh/Cr + PCr, GPC + PCh/Cr + PCr, and Ins/Cr + PCr  **DTI**  Brainstem  Reduced FA in the brainstem. | Correlations between RAVLT-coding and:  **Structure MRI**  *VBM*  Cerebrum  Right angular gyrus (+)  Right superior temporal gyrus (+)  Right superior frontal gyrus (+)  Left medial frontal gyrus (+)  Left insula (+)  Left medial temporal gyrus (+)  Right parahippocampal gyrus (+)  Cerebellum  Left culmen (+)  Correlations between RAVLT-delayed recall and:  **Structure MRI**  *VBM*  Cerebrum Right precentral gyrus (+)  Left insula (+)  Left inferior temporal gyrus (+)  Left superior temporal gyrus (+)  Left inferior parietal lobe (+)  Cerebellum  Bilateral culmen (+)  Correlations between RAVLT-recognition and:  **Structure MRI**  *VBM*  Cerebrum  Right inferior frontal gyrus (+)  Correlations between RPM and:  **Structure MRI**  *VBM*  Cerebrum  Right precuneus (+)  Left inferior temporal gyrus (+)  Cerebellum  Right cerebellar tonsil (+)  Correlations between CBTT (forward) and:  **Structure MRI**  *VBM*  Cerebrum  Left inferior parietal lobe (+)  **MRS**  Cerebellum  Glu (*r* = -.42)  Glx (*r* = -.41)  Correlations between DS (forward) and:  **Structure MRI**  *VBM*  Cerebrum  Left inferior temporal gyrus (+)  **MRS**  Cerebellum  NAA (*r* = .42)  NAA + NAAG (*r* = .43)  **DTI**  Brainstem  Brainstem (*r* = .49)  Correlations between semantic verbal fluency and:  **Structure MRI**  *VBM*  Cerebrum  Left precentral gyrus (+)  **MRS**  Cerebellum  PCh (*r* = .66)  GPC + PCh (*r* = .69) |
| (Chen et al., 2014)  Taiwan | Case control  Level: III-2 | SCA2: 5  SCA3: 18  SCA6: 3  MSA-C: 12  HC: 44 | Mean age:  SCA2: 60.6 ± 11.1  SCA3: 46.3 ± 10.0  SCA6: 59.0 ± 13.9  MSA-C: 60.6 ± 6.1  HC: 51.1 ± 17.9  Mean onset age: -  Disease duration (years):  SCA2: 3.7 ± 3.0  SCA3: 6.1 ± 4.0  SCA6: 6.8 ± 2.4  MSA-C: 4.3 ± 2.2  Gender (M/F):  -  CAG repeat length:  -  Education: - | 1.5 | SARA | MRS | Cerebellum  Reduced NAA/Cr and NAA/Cho in the cerebellar hemispheres.  Reduced NAA/Cr in the vermis. | - |
| (Kang et al., 2014)  Germany | Case control  Level: III-2 | SCA3: 12  HC: 12 | Mean age:  SCA3: 50.5 ± 10.4  HC: 47.8 ± 11.0  Mean onset age: -  Disease duration (years):  SCA3: 11.0 ± 6.4  Gender (M/F):  SCA3: 7/5  HC: 7/5  CAG repeat length:  SCA3: 70.6 ± 3.8  Education: - | 3.0 | SARA | Structure MRI   - VBM   DTI | **Structure MRI**  *VBM*  Cerebrum  Reduced GM and WM of bilateral thalamus (subcortical).  Brainstem  Reduced GM of brainstem, including pons and midbrain.  Reduced WM of pons and anterior midbrain.  Cerebellum  Reduced GM and WM of cerebellum.  **DTI**  Cerebrum  Reduced FA and increased MD in bilateral frontal, parietal, temporal, and occipital lobes (cortical), and thalamus (subcortical).  Brainstem  Reduced FA and increased MD in brainstem.  Cerebellum  Reduced FA and increased MD in cerebellum. | *Negative correlations between SARA and:  **DTI**  Cerebrum  Bilateral frontal lobe FA  Bilateral thalamus  Brainstem  Midbrain  Cerebellum  Cerebellum |
| (Adanyeguh et al., 2015)  France | Case control  Level: III-2 | SCA1: 16  SCA2: 12  SCA3: 21  SCA7: 12  HC: 33 | Mean age:  SCA1: 44.0 ± 16.0  SCA2: 45.0 ± 13.0  SCA3: 51.0 ± 12.0  SCA7: 46.0 ± 14.0  HC: 48.0 ± 13.0  Mean onset age: -  Disease duration (years):  SCA1: 7.0 ± 7.0  SCA2: 10.0 ± 6.0  SCA3: 9.0 ± 5.0  SCA7: 9.0 ± 5.0  Gender (M/F):  SCA1: 9/7  SCA2: 7/5  SCA3: 9/12  SCA7: 6/6  HC: 15/18  CAG repeat length:  SCA1: 47.0 ± 7.0  SCA2: 40.0 ± 3.0  SCA3: 69.0 ± 6.0  SCA7: 42.0 ± 5.0  Education: - | 3.0 | SARA | MRS | Brainstem  Reduced NAA and Glu increased Cr, myo-Ins in the pons.  Cerebellum  Reduced NAA and Glu Increased Cr, myo-Ins in the vermis. | Correlations between SARA and:  Brainstem  Pons   - Cr (*r* = .64) - myo-Ins (*r* = .69) - NAA (*r* = -.82)   *No correlation reported in vermis. |
| (de Rezende et al., 2015)  Brazil | Case control  Level: III-2 | SCA3: 49  HC: 49 | Mean age:  SCA3: 47.7 ± 13.0  HC: 47.5 ± 12.7  Mean onset age:  SCA3: 37.5 ± 12.5  Disease duration (years):  SCA3: 10.0 ± 4.7  Gender (M/F):  SCA3: 27/22  HC: 27/22  CAG repeat length:  SCA3: 72.1 ± 4.2  Education: - | 3.0 | SARA | Structure MRI   - Volumetric - Surface analysis | **Structure MRI**  *Volumetric*  Cerebrum  Atrophy in the bilateral hippocampi, caudate, putamen, pallidum, thalamus, and ventral diencephalon (subcortical).  Brainstem  Atrophy in the right brainstem.  Cerebellum  Atrophy in the GM and WM of bilateral cerebellum.  *Surface analysis*  Cerebrum  Reduced cortical thickness in the left superior frontal, superior temporal, and precentral cortices; and right superior frontal cortex (cortical). | Correlations between SARA and:  **Structure MRI**  *Volumetric*  Cerebrum  Left thalamus (*r* = .62)  Right ventral diencephalon (*r* = .58)  Left ventral diencephalon (*r* = .64)  Brainstem  Brainstem (*r* = .58)  *Surface analysis*  Cerebrum  Left precentral gyrus (*r* = -.30)  Anterior transverse temporal gyrus (*r* = -.30)  Superior temporal sulcus (*r* = -.35)  Caudal middle frontal cortex (*r* = -.33)  Paracentral cortex (*r* = -.31)  Transverse temporal cortex (*r* = -.35)  Correlations between Similarities and:  **Structure MRI**  *Surface analysis*  Cerebrum  Left precentral gyrus (*r* = .82)  Right superior occipital gyrus (*r* = .84)  Correlation between RPM and:  **Structure MRI**  *Surface analysis*  Cerebrum  Left middle occipital gyrus (*r* = .90) |
| (Nunes et al., 2015)  Brazil | Case control  Level: III-2 | SCA3 (dystonia): 21 (19 MRI)  SCA (no dystonia): 54 (33 MRI)  HC: 52 | Mean age:  SCA3 (dystonia): 39.7 ± 13.9  SCA3 (no dystonia): 50.0 ± 11.1  Mean onset age:  SCA3 (dystonia): 28.9 ± 11.7  SCA3 (no dystonia): 40.6 ± 11.7  Disease duration (years): -  Gender (M/F):  SCA3: 13/8  HC: 25/29  CAG repeat length:  SCA3 (dystonia): 75.3 ± 3.6  SCA3 (no dystonia): 70.9 ± 3.0  Education: - | 3.0 | BMFDRS | Structure MRI   - Volumetric - Surface analysis   DTI | **Structural MRI**  *Volumetric*  Cerebrum  Atrophy in the bilateral hippocampi, caudate, putamen, pallidum, thalamus, and ventral diencephalon (subcortical).  *Greater atrophy in thalami and ventral diencephalic in dystonia group.  Brainstem  Atrophy in the right brainstem.  Cerebellum  Atrophy in the GM and WM of bilateral cerebellum.  *Greater atrophy in WM of cerebellum in dystonia group.  *Surface analysis*  Cerebrum  *Reduced cortical thickness in pre- and paracentral cortices (cortical; dystonia).  *Reduced cortical thickness in occipital lobes (cortical; no dystonia). | No correlation between MRI modalities with BMFDRS scores. |
| (Hernandez-Castillo et al., 2017)  Mexico | Case control  Level: III-2 | SCA3: 17  HC: 17 | Mean age:  SCA3: 40.1 ± 11.9  Mean onset age:  SCA3: 33.5 ± 9.8  Disease duration (years):  SCA3: 6.9 ± 4.5  Gender (M/F):  SCA3: 7/10  HC: 7/10  CAG repeat length: -  Education: - | 3.0 | SARA | Structure MRI   - VBM | Cerebrum  Reduced GM of the occipital cortex (left lingual gyrus; cortical).  Brainstem  Reduced GM of the right brainstem and pons.  Cerebellum  Reduced GM in the bilateral Crus II, left lobule I-IV, right lobule V, and vermis IX. | *Negative correlations between SARA scores and:  Cerebrum  Right paracentral lobule  Cerebellum  Right declive lobule VI  Left culmen lobule VI  Left tonsil lobule IX |
| (Wu et al., 2017)  China | Case control  Level: III-2 | SCA3: 22  Pre-SCA3: 16  HC: 24 | Mean age:  SCA3: 43.4 ± 5.9  Pre-SCA3: 28.8 ± 7.2  Mean onset age:  SCA3: -  Disease duration (years):  SCA3: 7.0 ± 4.4  Gender (M/F):  SCA3: 14/8  Pre-SCA3: 5/11  CAG repeat length:  SCA3: -  Education: - | 3.0 | ICARS  SARA | DTI | Cerebrum  Increased MD in the corpus callosum, internal capsule, external capsule, fornix, superior corona radiata, posterior thalamic radiation, sagittal stratum, and superior fronto-occipital fasciculus (subcortical).  Brainstem  Reduced FA in the pontine crossing tract and bilateral lemniscus.  Cerebellum  Reduced FA and increased MD in the cerebellar peduncle. | *Negative correlations between ICARS and FA:  Common WM skeleton (*r* = -.54)  Brainstem  Medial lemniscus (*r* = -.52)  Cerebellum  Right superior cerebellar peduncle (*r* = -.43)  *Positive correlations between ICARS and MD:  Middle cerebellar peduncle (*r* = .48)  Left anterior limb of internal capsule (*r* = .45)  Left superior corona radiata (*r* = .61)  External capsule (*r* = .50)  *Correlation between SARA and FA:  Cerebrum  Right posterior thalamic radiation (*r* = .44) |
| (Rezende et al., 2018)  Brazil | Case control  Level: III-2 | SCA3: 79  Pre-SCA3: 12  HC: 91 | Mean age:  SCA3 (Total): 48.4 ± 12.6  HC: 47.9 ± 12.4  Mean onset age:  SCA3: -  Disease duration (years):  SCA3: 10.4 ± 6.8  Gender (M/F):  SCA3: 40/39  Pre-SCA: 2/10  HC: 42/50  CAG repeat length:  SCA3: 71.9 ± 3.7  Pre-SCA3: 69.5 ± 2.5  Education: - | 3.0 | SARA | Structure MRI   - Volumetric - Surface analysis   DTI | **Structure MRI**  *Volumetric*  Cerebrum  Atrophy in the bilateral caudate, globus pallidum, and thalami (subcortical).  Brainstem  Atrophy in the red nuclei, medulla, midbrain, substantia nigra, and pons.  Cerebellum  Atrophy in the bilateral Crus I and II, lobules I-IV, lobule VIIb, lobule VIIIa and VIIIb, lobule X, vermix IX, left lobules V and VI.  Spinal cord  Atrophy in the cervical spinal cord.  Increased eccentricity in the cervical spinal cord.  *Surface analysis*  Cerebrum  Reduced cortical thickness in the superior temporal sulci, left precentral gyrus, inferior precentral gyrus, middle-posterior cingulate gyri and sulci, paracentral gyri and sulci, and right central sulcus.  **DTI**  Cerebrum  Decreased FA in superior parietal gyri, precentral gyri, postcentral gyri, middle frontal gyri, right middle occipital gyrus, left lingual gyrus, and right cuneus (cortical), and bilateral posterior thalamic radiation, bilateral corona radiata, limb of internal capsule, and corpus callosum (subcortical).  Increased MD and RD in cerebral peduncles and corpus callosum (subcortical).  Brainstem  Increased AD in bilateral medial lemniscus, bilateral cortical-spinal tract, midbrain, and medulla.  Decreased FA and increased MD in middle lemnisci and cortical-spinal tract.  Increased RD in pyramidal tracts.  Cerebellum  Increased AD in bilateral superior, middle, and inferior, and whole cerebellar peduncle.  Decreased FA and increased MD and RD in cerebellar peduncles. | - |
| (Jao et al., 2019a)  Taiwan | Case control  Level: III-2 | SCA3: 15  MSA-C: 15  HC: 30 | Mean age:  SCA3: 43.8 ± 14.8  MSA-C: 56.2 ± 6.6  HC: 46.6 ± 16.2  Mean onset age:  SCA3: -  Disease duration (years): -  Gender (M/F):  SCA3: 7/8 MSA-C: 7/8  HC: 15/15  CAG repeat length: -  Education: - | 1.5 | - | DTI | Cerebrum  Decreased FA and increased MD and RD in the posterior limb of internal capsule, anterior corona radiates, and external capsule (subcortical); and frontal and temporal lobes (cortical).  Brainstem  Decreased FA and increased MD and RD in the cortical-spinal tract.  Cerebellum  Decreased FA and increased MD and RD in the middle and inferior cerebellar peduncle. | - |
| (Jao et al., 2019b)  Taiwan | Case control  Level: III-2 | SCA3: 48  HC: 48 | Mean age:  SCA3: 48.1 ± 11.8  HC: 48.1 ± 12.1  Mean onset age:  SCA3: -  Disease duration (years):  SCA3: 8.9 ± 6.4  Gender (M/F):  SCA3: 27/21  HC: 24/24  CAG repeat length: -  Education: - | 1.5 | SARA | Structure MRI   - Volumetric | **Structure MRI**  *Volumetric*  Cerebrum  Atrophy in the frontal, parietal, temporal and occipital lobes (cortical); and limbic system, caudate nucleus, lenticular nucleus, and putamen.  *Mainly left lateralized, especially temporal and occipital lobes.  Cerebellum  Atrophy in the cerebellar hemisphere, vermis, and whole cerebellum.  *3D-FD analysis*  Cerebellum  *Dissociated from higher level brain networks.  Network  *Decreased intra-modular connectivity in all lobes.  *Increased inter-modular connectivity in the frontal and parietal lobes. | - |
| (Peng et al., 2019)  China | Case control  Level: III-2 | SCA3: 31  HC: 31 | Mean age:  SCA3: 38.9 ± 7.4  HC: 37.5 ± 10.2  Mean onset age:  SCA3: 34.9 ± 6.6  Disease duration (years):  SCA3: 4.8 ± 3.6  Gender (M/F):  SCA3: 15/16  HC: 15/16  CAG repeat length:  SCA3: 71.8 ± 2.6  Education: - | 1.5 | ICARS | Structure MRI   - VBM   MRS  DTI | **Structure MRI**  *VBM*  Cerebrum  Reduced GM in bilateral inferior frontal gyrus, insula, and left superior frontal gyrus (cortical).  Brainstem  Reduced GM in the pons, midbrain, and medulla.  Cerebellum  Reduced GM in the bilateral cerebellar culmen.  **MRS**  Cerebrum  Reduced NAA/Cr and NAA/Cho in the thalamus.  *No change in putamen.  Cerebellum  Reduced NAA/Cr and | Correlations between ICARS and:  **Structure MRI**  *VBM (GM)*  Brainstem  Right pons (-)  Left midbrain (-)  Cerebellum  Left cerebellar culmen (-)  Left cerebellar lingual (-)  **MRS**  Cerebellum  Middle cerebellar peduncle   - NAA/Cr (*r* = -.45)   Dentate nucleus   - NAA/Cr (*r* = -.50) - Cho/Cr (*r* = -.37) |
|  |  |  |  |  |  |  | NAA/Cho in middle cerebellar peduncle, dentate nucleus, and vermis.  Reduced NAA/Cr in the cerebellar cortex.  Reduced Cho/Cr in the cerebellar vermis.  **DTI**  Cerebellum  Reduced FA and increased MD in the superior, middle, and inferior cerebellar peduncles. | **DTI**  Cerebellum  Superior cerebellar peduncle   - FA (*r* = -.64) - MD (*r* = .56)   Middle cerebellar peduncle   - FA (*r* = -.42) - MD (*r* = .44)   Inferior cerebellar peduncle   - FA (*r* = -.60) |
| (Arruda et al., 2020)  Brazil | Case control  Level: III-2 | SCA3: 19  HC(SCA3):19  SCA10: 18  HC (SCA10): 18 | Mean age:  SCA3: 46.0 ± 12.5  HC(SCA3): 45.0 ± 11.7  SCA10: 46.4 ± 8.0  HC(SCA10): 47.1 ± 8.9  Mean onset age:  SCA3: 34.5 ± 8.4  SCA10: 32.7 ± 8.5  Disease duration (years):  SCA3: 11.6 ± 6.6  SCA10: 13.2 ± 11.5  Gender (M/F):  SCA3: 11/8  HC(SCA3): 11/8  SCA10: 9/9  HC(SCA10): 9/9  CAG repeat length:  SCA3: 71.5 ± 4.6  SCA10: 10.4 ± 6.2  Education: - | 3.0 | SARA | Structure MRI   - Surface analysis | Cerebrum  Reduced cortical thickness in the right caudal middle frontal gyrus. Left pars triangularis of the inferior frontal gyrus, right temporal pole and middle temporal gyrus. | Correlation between SARA scores and:  Cerebellum  WM (*r* = -.46)  *No correlation with thalamus, pallidum, brainstem, subcortical GM, cerebellar GM, and total GM. |
| (Guo et al., 2020)  China | Case control  Level: III-2 | SCA3: 47  HC: 49 | Mean age:  SCA3: 39.9 ± 11.9  HC: 41.3 ± 12.4  Mean onset age:  SCA3: -  Disease duration (years):  SCA3: 6.7 ± 4.4  Gender (M/F):  SCA3: 28/19  HC: 25/24  CAG repeat length:  SCA3: 65.9 ± 3.5  Education: - | 3.0 | SARA | Structure MRI   - VBM | Cerebrum  Reduced GM in right inferior frontal gyrus, right parietal lobe, motor and association cortex (cortex); and caudate and putamen (subcortical).  Cerebellum  Reduced GM in the vermis, cerebellar hemispheres.  *Degeneration starts from vermis, followed by cerebellar hemispheres, then cerebral cortex and finally cerebral subcortical structures. | - |
| (Meira et al., 2020)  Brazil | Case control  Level: III-2 | SCA3: 19  HC(SCA3):19  SCA10: 18  HC (SCA10): 18 | Mean age:  SCA3: 44.8 ± 12.5  HC(SCA3): 45.0 ± 11.7  SCA10: 48.9 ± 7.5  HC(SCA10): 48.3 ± 8.4  Mean onset age:  SCA3: 34.2 ± 8.4  SCA10: 33.8 ± 8.1  Disease duration (years):  SCA3: 11.9 ± 7.5  SCA10: 11.3 ± 10.2  Gender (M/F):  SCA3: 11/8  HC(SCA3): 11/8  SCA10: 9/9  HC(SCA10): 9/9  CAG repeat length:  SCA3: 71.7 ± 5.0  SCA10: 1918 ± 185.2  Education: - | 3.0 | SARA | DTI | Cerebrum  Reduced FA in forceps major, forceps minor, right cingulate fasciculus, parietal and temporal superior longitudinal fasciculus, left anterior thalamic radiation, and bilateral inferior longitudinal fasciculus.  Brainstem  Reduced FA in the bilateral cortical-spinal tract. | Correlation between SARA scores and:  Brainstem  Left cortical-spinal tract   - FA (*r* = -.47)   Right cortical-spinal tract   - FA (*r* = -.51) |
| (Inada et al., 2021)  Brazil | Case control  Level: III-2 | Cohort 1  SCA3: 29 HC: 29  Cohort 2  SCA3: 91  HC: 91 | Cohort 1  Mean age:  SCA3: 40.5 ± 10.5  HC: 42.5 ± 9.6  Mean onset age: -  Disease duration (years):  SCA3: 7.1 ± 4.2  Gender (M/F):  SCA3: 13/16  HC: 13/16  CAG repeat length:  SCA3: 71.0 ± 4.2  Education: -  Cohort 2  Mean age:  SCA3: 48.2 ± 12.8  HC: 47.9 ± 12.5  Mean onset age: -  Disease duration (years):  SCA3: 10.4 ± 6.8  Gender (M/F):  SCA3: 42/49  HC: 42/49  CAG repeat length:  SCA3: 71.9 ± 3.7  Education: - | **Cohort 1:**  1.5  **Cohort 2:**  3.0 | SARA | DTI | **Cohort 1**  Cerebrum  Reduced FA in the bilateral precentral gyrus.  *No change in superior corona radiata and posterior limb of internal capsule.  Brainstem  *No change in basis pontis.  Cerebellum  *No change in cerebellar peduncle.  **Cohort 2**  Cerebrum  Reduced FA in the bilateral precentral gyrus, superior corona radiata, and posterior limb of internal capsule.  Increased MD & RD in the left precentral gyrus, bilateral superior corona radiata and posterior limb of internal capsule.  Increased AD in the bilateral posterior limb of internal capsule.  Brainstem  Reduced FA and increased MD, RD & AD in the bilateral basis pontis.  Cerebellum  Reduced FA and increased MD & RD in the bilateral cerebellar peduncle.  Increased AD in the left cerebellar peduncle. | **Cohort 1**  No correlation between DTI values with SARA scores.  **Cohort 2**  Correlations between SARA scores and:  Cerebrum  Precentral gyrus   - FA (-; moderate) - MD (+; moderate) - RD (+; moderate) - AD (+ weak)   Superior corona radiata   - FA (-; moderate) - MD (+; moderate) - RD (+; moderate) - AD (+ weak)   Brainstem  Basis pontis   - FA (-; moderate) - MD (+; moderate) - RD (+; moderate) - AD (+ weak)   Cerebellum  Cerebellar peduncle   - FA (-; moderate) - MD (+; moderate) - RD (+; moderate) - AD (+ weak) |

_*Abbreviation:_ _AD, Axial diffusivity; BDI, Beck Depression Index; BMFDRS, Burke–Marsden–Fahn's Dystonia Rating Scale; BNT, Boston Naming Test; CBTT, Corsi-Block Tapping Test; Cho, Choline; Cr, Creatinine; DRPLA, DS, Digit Span; DTI, Diffusion tensor imaging; FA, Fractional anisotropy; GM, Grey matter; HC, Healthy control; ICARS, International Cooperative Ataxia Rating Scale; LM, Logical Memory; MD, Mean diffusivity; MRI, Magnetic resonance imaging; MRS, Magnetic resonance spectroscopy; MSA-C, Multiple system atrophy – cerebellum type; myo-Ins; myo-Isonitol; NAA, N-Acetylaspartate; PC, Picture Completion; RAVLT, Rey Auditory Verbal Learning Test; RD, Radial diffusivity; RPM, Raven’s Progressive Matrices; SARA, Scale for the Assessment and Rating of Ataxia; SCA, Spinocerebellar ataxia; UHDRS, Unified Huntington's Disease Rating Scale; VBM, Voxel-based morphometry; VPA, Visual Paired Associates; WCST, Wisconsin Card Sorting Test; WM, White matter._

Adanyeguh, I.M., Henry, P.G., Nguyen, T.M., Rinaldi, D., Jauffret, C., Valabregue, R., Emir, U.E., Deelchand, D.K., Brice, A., and Eberly, L.E. (2015). In vivo neurometabolic profiling in patients with spinocerebellar ataxia types 1, 2, 3, and 7. *Movement Disorders* 30**,** 662-670.

Arruda, W.O., Meira, A.T., Ono, S.E., De Carvalho Neto, A., Betting, L.E.G.G., Raskin, S., Camargo, C.H.F., and Teive, H.a.G. (2020). Volumetric MRI changes in spinocerebellar ataxia (SCA3 and SCA10) patients. *The Cerebellum* 19**,** 536-543.

Bürk, K., Abele, M., Fetter, M., Dichgans, J., Skalej, M., Laccone, F., Didierjean, O., Brice, A., and Klockgether, T. (1996). Autosomal dominant cerebellar ataxia type I clinical features and MRI in families with SCA1, SCA2 and SCA3. *Brain* 119**,** 1497-1505.

Camargos, S.T., Marques-Jr, W., and Santos, A.C.D. (2011). Brain stem and cerebellum volumetric analysis of Machado Joseph disease patients. *Arquivos de Neuro-Psiquiatria* 69**,** 292-296.

Chen, H.-C., Lirng, J.-F., Soong, B.-W., Guo, W.Y., Wu, H.-M., Chen, C.C.-C., and Chang, C.-Y. (2014). The merit of proton magnetic resonance spectroscopy in the longitudinal assessment of spinocerebellar ataxias and multiple system atrophy-cerebellar type. *Cerebellum & ataxias* 1**,** 1-10.

D’abreu, A., França Jr, M.C., Yasuda, C.L., Campos, B.A., Lopes‐Cendes, I., and Cendes, F. (2012). Neocortical atrophy in Machado‐Joseph disease: A longitudinal neuroimaging study. *Journal of Neuroimaging* 22**,** 285-291.

D’abreu, A., França Jr, M.C., Yasuda, C.L., Souza, M.S., Lopes‐Cendes, Í., and Cendes, F. (2011). Thalamic volume and dystonia in Machado–Joseph disease. *Journal of Neuroimaging* 21**,** e91-e93.

De Rezende, T., D'abreu, A., Guimarães, R., Lopes, T., Lopes‐Cendes, I., Cendes, F., Castellano, G., and França Jr, M. (2015). Cerebral cortex involvement in Machado−Joseph disease. *European Journal of Neurology* 22**,** 277-e224.

Etchebehere, E.C., Cendes, F., Lopes-Cendes, I., Pereira, J.A., Lima, M.C., Sansana, C.R., Silva, C.A., Camargo, M.F., Santos, A.O., and Ramos, C.D. (2001). Brain single-photon emission computed tomography and magnetic resonance imaging in Machado-Joseph disease. *Archives of Neurology* 58**,** 1257-1263.

Guimarães, R.P., D'abreu, A., Yasuda, C.L., França Jr, M.C., Silva, B.H., Cappabianco, F.A., Bergo, F.P., Lopes‐Cendes, I.T., and Cendes, F. (2013). A multimodal evaluation of microstructural white matter damage in spinocerebellar ataxia type 3. *Movement Disorders* 28**,** 1125-1132.

Guo, J., Chen, H., Biswal, B.B., Guo, X., Zhang, H., Dai, L., Zhang, Y., Li, L., Fan, Y., and Han, S. (2020). Gray matter atrophy patterns within the cerebellum-neostriatum-cortical network in SCA3. *Neurology* 95**,** e3036-e3044.

Hernandez-Castillo, C.R., Diaz, R., Campos-Romo, A., and Fernandez-Ruiz, J. (2017). Neural correlates of ataxia severity in spinocerebellar ataxia type 3/Machado-Joseph disease. *Cerebellum & Ataxias* 4**,** 1-4.

Inada, B.S.Y., Rezende, T.J.R., Pereira, F.V., Garcia, L.Á.L., Da Rocha, A.J., Neto, P.B., Barsottini, O.G.P., França Jr, M.C., and Pedroso, J.L. (2021). Corticospinal tract involvement in spinocerebellar ataxia type 3: A diffusion tensor imaging study. *Neuroradiology* 63**,** 217-224.

Jao, C.-W., Soong, B.-W., Huang, C.-W., Duan, C.-A., Wu, C.-C., Wu, Y.-T., and Wang, P.-S. (2019a). Diffusion tensor magnetic resonance imaging for differentiating multiple system atrophy cerebellar type and spinocerebellar ataxia type 3. *Brain Sciences* 9**,** 354.

Jao, C.-W., Soong, B.-W., Wang, T.-Y., Wu, H.-M., Lu, C.-F., Wang, P.-S., and Wu, Y.-T. (2019b). Intra-and inter-modular connectivity alterations in the brain structural network of spinocerebellar ataxia type 3. *Entropy* 21**,** 317.

Kang, J.-S., Klein, J., Baudrexel, S., Deichmann, R., Nolte, D., and Hilker, R. (2014). White matter damage is related to ataxia severity in SCA3. *Journal of Neurology* 261**,** 291-299.

Lei, L., Liao, Y., Liao, W., Zhou, J., Yuan, Y., Wang, J., Jiang, H., Shen, L., and Tang, B. (2011). Magnetic resonance spectroscopy of the cerebellum in patients with spinocerebellar ataxia type 3/Machado-Joseph disease. *Zhong nan da xue xue bao. Yi xue ban= Journal of Central South University. Medical Sciences* 36**,** 511-519.

Liang, X., Jiang, H., Chen, C., Zhou, G., Wang, J., Zhang, S., Lei, L., Wang, X., and Tang, B. (2009). The correlation between magnetic resonance imaging features of the brainstem and cerebellum and clinical features of spinocerebellar ataxia 3/Machado-Joseph disease. *Neurology India* 57**,** 578.

Lirng, J.-F., Wang, P.-S., Chen, H.-C., Soong, B.-W., Guo, W.Y., Wu, H.-M., and Chang, C.-Y. (2012). Differences between spinocerebellar ataxias and multiple system atrophy-cerebellar type on proton magnetic resonance spectroscopy. *PLoS One* 7**,** e47925.

Lopes, T.M., Anelyssa, D., Junior, M.C.F., Yasuda, C.L., Betting, L.E., Samara, A.B., Castellano, G., Somazz, J.C., Balthazar, M.L.F., and Lopes-Cendes, I. (2013). Widespread neuronal damage and cognitive dysfunction in spinocerebellar ataxia type 3. *Journal of Neurology* 260**,** 2370-2379.

Meira, A.T., Arruda, W.O., Ono, S.E., Franklin, G.L., De Carvalho Neto, A., Raskin, S., Ashizawa, T., Camargo, C.H.F., and Teive, H.A. (2020). Analysis of diffusion tensor parameters in spinocerebellar ataxia type 3 and type 10 patients. *Parkinsonism & Related Disorders* 78**,** 73-78.

Nunes, M.B., Martinez, A.R.M., Rezende, T.J.R., Friedman, J.H., Lopes-Cendes, I., D'abreu, A., and França Jr, M.C. (2015). Dystonia in Machado–Joseph disease: Clinical profile, therapy and anatomical basis. *Parkinsonism & Related Disorders* 21**,** 1441-1447.

Ogawa, Y., Ito, S., Makino, T., Kanai, K., Arai, K., and Kuwabara, S. (2012). Flattened facial colliculus on magnetic resonance imaging in Machado–Joseph disease. *Movement Disorders* 27**,** 1041-1046.

Peng, H., Liang, X., Long, Z., Chen, Z., Shi, Y., Xia, K., Meng, L., Tang, B., Qiu, R., and Jiang, H. (2019). Gene-related cerebellar neurodegeneration in SCA3/MJD: A case-controlled imaging-genetic study. *Frontiers in Neurology* 10**,** 1025.

Rezende, T.J.R., De Paiva, J.L.R., Martinez, A.R.M., Lopes‐Cendes, I., Pedroso, J.L., Barsottini, O.G.P., Cendes, F., and França Jr, M.C. (2018). Structural signature of SCA3: From presymptomatic to late disease stages. *Annals of Neurology* 84**,** 401-408.

Schulz, J.B., Borkert, J., Wolf, S., Schmitz-Hübsch, T., Rakowicz, M., Mariotti, C., Schoels, L., Timmann, D., Van De Warrenburg, B., and Dürr, A. (2010). Visualization, quantification and correlation of brain atrophy with clinical symptoms in spinocerebellar ataxia types 1, 3 and 6. *Neuroimage* 49**,** 158-168.

Wang, P.-S., Chen, H.-C., Wu, H.-M., Lirng, J.-F., Wu, Y.-T., and Soong, B.-W. (2012). Association between proton magnetic resonance spectroscopy measurements and CAG repeat number in patients with spinocerebellar ataxias 2, 3, or 6. *PLoS One* 7**,** e47479.

Wu, X., Liao, X., Zhan, Y., Cheng, C., Shen, W., Huang, M., Zhou, Z., Wang, Z., Qiu, Z., and Xing, W. (2017). Microstructural alterations in asymptomatic and symptomatic Patients with spinocerebellar ataxia Type 3: A tract-based spatial statistics study. *Frontiers in Neurology* 8**,** 714-722.

Yoshizawa, T., Watanabe, M., Frusho, K., and Shoji, S. (2003). Magnetic resonance imaging demonstrates differential atrophy of pontine base and tegmentum in Machado–Joseph disease. *Journal of the Neurological Sciences* 215**,** 45-50.
