# Supplementary material for: Magnetic Resonance Imaging and Its Clinical Correlation in Spinocerebellar Ataxia Type 3: A Systematic Review
Source: Front Neurosci. 2022 Jun 10;16:859651. doi: 10.3389/fnins.2022.859651 (PMC9226753; doi:10.3389/fnins.2022.859651)
Supplement: Supplementary file 2 [file Table_2.docx]

Supplementary Table 3

*Summary of MRI sequences and findings for included studies.*

| **First author, year** | **Cerebrum (cortical)** | **Cerebrum (subcortical)** | **Brainstem** | **Cerebellum** |
| --- | --- | --- | --- | --- |
| (Bürk et al., 1996) |  |  | Structural (Volumetric)  Brainstem   - ↓Pons - ↓Medulla | Structural (Volumetric)  ↓Vermis  ↓Middle cerebellar peduncles |
| (Etchebehere et al., 2001) |  |  |  | Structural (Volumetric)  ↓Cerebellar hemispheres  ↓Vermis |
| (Yoshizawa et al., 2003) |  |  | Structural (Volumetric)  Brainstem   - ↓Pons |  |
| (Liang et al., 2009) |  |  | Structural (Volumetric)  ↓ Brainstem   - - Midbrain | Structural (Volumetric)  ↓ Cerebellum |
| (Schulz et al., 2010) | Structural (Volumetric)  ↓ Temporal  - Frontal  - Parietal  - Occipital | Structural (Volumetric)  Basal ganglia   - ↓ Putamen - ↓ Caudate | Structural (Volumetric)  ↓ Brainstem   - ↓ Pons - ↓ Medulla - ↓ Midbrain | Structural (Volumetric)  ↓ Cerebellar hemispheres  ↓ Vermis |
|  | Structural (VBM: GM)   - Cerebral hemispheres | Structural (VBM: GM)  - Basal ganglia | Structural (VBM: GM)  - Pons | Structural (VBM: GM)  ↓Cerebral hemispheres  ↓Vermis |
|  |  |  | Structural (VBM: WM)  ↓ Brainstem   - ↓ Pons - ↓ Midbrain | Structural (VBM: WM)  ↓Cerebral hemispheres  ↓ Cerebellar peduncles |
| (Camargos et al., 2011) |  |  | Structural (Volumetric)↓Brainstem   - ↓Pons - ↓Medulla - ↓Midbrain | Structural (Volumetric)  ↓Cerebellum  ↓Cerebellar hemispheres   - - Tonsil   ↓Vermis |
| (D’Abreu et al., 2011) |  | Structural (Volumetric)  Diencephalon   - ↓ Thalamus |  |  |
| (Lei et al., 2011) |  |  |  | MRS (NAA/Cr)  ↓ Cerebellar hemispheres  ↓ Vermis   - ↓Dentate   ↓ Middle cerebellar peduncles  MRS (NAA/Cho)  ↓Vermis   - ↓Dentate |
| (D’Abreu et al., 2012) | Structural (VBM: GM)  ↓Frontal  ↓Parietal  ↓Temporal  ↓Occipital | Structural (VBM: GM)  Basal ganglia   - ↓Lentiform nucleus - ↓ Caudate   ↓ Limbic lobe  ↓ Claustrum | Structural (VBM: GM)  ↓ Brainstem   - ↓ Pons - ↓ Medulla - ↓ Midbrain | Structural (VBM: GM)  ↓ Cerebellar hemispheres  ↓Vermis |
| (Lirng et al., 2012) |  |  |  | MRS (NAA/Cr & NAA/Cho)  ↓ Cerebellar hemispheres  ↓ Vermis  MRS (Cho/Cr)  - Cerebellum |
| (Ogawa et al., 2012) |  |  | Structural (Volumetric)  ↓Facial colliculus | Structural (Volumetric)  ↓Superior cerebellar peduncles |
| (Wang et al., 2012) |  |  |  | MRS (NAA/Cr)  ↓ Cerebellar hemispheres  ↓ Vermis |
| (Guimarães et al., 2013) |  | Structural (VBM: GM)  ↓ Limbic lobe | Structural (VBM: GM)  Brainstem   - ↓ Pons - ↓ Medulla   ↓ Pyramids | Structural (VBM: GM)  ↓ Cerebellum  Cerebellar hemispheres   - ↓ Tonsil - ↓ Inferior semilunar lobule   ↓ Vermis   - ↓Declive - ↓Uvula - ↓Fastigium - ↓ Tuber |
|  |  |  | Structural (VBM: WM)  ↓ Brainstem   - ↓ Medulla   ↓ Pyramids | Structural (VBM: WM)  ↓ Cerebellum  ↓ Vermis   - ↓Declive - ↓Uvula - ↓Fastigium - ↓ Tuber   ↓Cerebellar peduncles |
| (Lopes et al., 2013) | Structural (VBM: GM)  ↓ Frontal  ↓ Parietal | Structural (VBM: GM)  Basal ganglia   - ↓ Putamen   Limbic lobe   - ↓Posterior cingulate |  |  |
|  |  |  |  | MRS (NAA/Cr + PCr, NAA + NAAG/Cr + PCr, and Glx/Cr + PCr)  ↓Cerebellum  MRS (Glu/Cr + PCr, PCh/Cr + PCr, GPC + PCh/Cr + PCr, and Ins/Cr + PCr)  - Cerebellum |
|  |  |  | DTI (FA)  ↓ Brainstem |  |
| (Chen et al., 2014) |  |  |  | MRS (NAA/Cr & NAA/Cho)  ↓ Cerebellar hemispheres  ↓ Vermis  MRS (Cho/Cr)  - Cerebellar hemispheres  - Vermis |
| (Kang et al., 2014) |  | Structural (VBM: GM & WM)  Diencephalon   - ↓Thalamus | Structural (VBM: GM & WM)  ↓ Brainstem   - ↓Midbrain - ↓Pons | Structural (VBM: GM & WM)  ↓Cerebellum |
|  | DTI (FA)  ↓Frontal  ↓Parietal  ↓Temporal  ↓Occipital | DTI (FA)  Diencephalon   - ↓Thalamus | DTI (FA)  ↓ Brainstem | DTI (FA)  ↓Cerebellum |
| (Adanyeguh et al., 2015) |  |  | MRS (NAA & Glu)  ↓Pons  MRS (Cr, myo-Ins)  ↑Pons | MRS (NAA, Glu, Cr, myo-Ins)  ↓Vermis |
| (de Rezende et al., 2015) |  | Structural (Volumetric)  Diencephalon   - ↓ Ventral diencephalon - ↓Thalamus   ↓Limbic lobe   - ↓Hippocampus   Basal ganglia   - ↓Globus pallidus - ↓Putamen - ↓Caudate | Structural (Volumetric)  ↓Brainstem | Structural (Volumetric)  ↓Cerebellum |
|  | Structural (Surface analysis)  ↓Frontal  ↓Temporal |  |  |  |
| (Nunes et al., 2015) |  | Structural (Volumetric)  Diencephalon   - ↓ Ventral diencephalon - ↓Thalamus   Limbic system   - ↓Hippocampus   Basal ganglia   - ↓Globus pallidus - ↓Putamen - ↓Caudate | Structural (Volumetric)  ↓Brainstem | Structural (Volumetric)  ↓Cerebellum |
|  | Structural (Surface analysis)  ↓Frontal  ↓Occipital |  |  |  |
| (Hernandez-Castillo et al., 2017) | Structural (VBM: GM)  ↓ Occipital |  | Structural (VBM: GM)  ↓ Brainstem   - ↓ Pons | Structural (VBM: GM)  ↓ Cerebellar hemispheres  ↓ Vermis |
| (Wu et al., 2017) |  |  | DTI (FA)  ↓Pons crossing tract  ↓Lemniscus | DTI (FA)  ↓ Cerebellar peduncles |
| (Rezende et al., 2018) |  | Structural (Volumetric)  Diencephalon   - ↓ Thalamus   Basal ganglia   - ↓ Globus pallidus - ↓ Putamen - ↓ Caudate - ↓ Substantia nigra | Structural (Volumetric)  Brainstem   - ↓Pons - ↓Medulla - ↓Midbrain | Structural (Volumetric)  ↓Cerebellar hemispheres  ↓Vermis |
|  | Structural (Surface analysis)  ↓Frontal  ↓Temporal | Structural (Surface analysis)  Limbic system   - ↓Posterior cingulate |  |  |
|  | DTI (FA)  ↓Frontal  ↓Parietal  ↓Occipital | DTI (FA)  ↓Thalamic radiation  ↓Corona radiata  ↓Corpus callosum  ↓Internal capsule | DTI (FA)  ↓Lemniscus  ↓Cortical-spinal tract | DTI (FA)  ↓Cerebellar peduncles |
| (Jao et al., 2019a) | DTI (FA)  ↓Frontal  ↓Parietal | DTI (FA)  ↓ Corona radiata  ↓ Internal capsule  ↓ External capsule | DTI (FA)  ↓ Cortical-spinal tract | DTI (FA)  ↓ Middle cerebellar peduncles  ↓ Inferior cerebellar peduncles |
| (Jao et al., 2019b) | Structural (Volumetric)  ↓Frontal  ↓Parietal  ↓Temporal  ↓Occipital | Structural (Volumetric)  Limbic system   - ↓Limbic lobe   Basal ganglia   - ↓Putamen - ↓Caudate   ↓Lenticular fasciculus |  | Structural (Volumetric)  ↓Cerebellum  ↓Cerebellar hemispheres  ↓Vermis |
| (Peng et al., 2019) | Structural (VBM: GM)  ↓Frontal  ↓Temporal |  | Structural (VBM:GM)  Brainstem   - ↓Pons - ↓Medulla - ↓Midbrain |  |
|  |  | MRS (NAA/Cr)  Diencephalon   - ↓Thalamus   Basal ganglia   - - Putamen |  | MRS (NAA/Cr)  ↓Cerebellar hemispheres  ↓Vermis   - ↓Dentate   ↓Middle cerebellar peduncles  MRS (NAA/Cho)  ↓Vermis  ↓Middle cerebellar peduncles  MRS (Cho/Cr)  ↓Vermis |
|  |  |  |  | DTI (FA)  ↓ Superior cerebellar peduncles  ↓ Middle cerebellar peduncles  ↓ Inferior cerebellar peduncles |
| (Arruda et al., 2020) | Structural (Surface analysis)  ↓Frontal  ↓Temporal |  |  |  |
| (Guo et al., 2020) | Structural (VBM:GM)  ↓Frontal  ↓Parietal | Structural (VBM:GM)  Basal ganglia   - ↓Putamen - ↓Caudate |  |  |
| (Meira et al., 2020) |  | DTI (FA)  ↓ Thalamic radiation  ↓ Forceps  ↓ Cingulate fasciculus  ↓ Parietal-temporal superior longitudinal fasciculus  ↓ Inferior longitudinal fasciculus | DTI (FA)  ↓ Cortical-spinal tract |  |
| (Inada et al., 2021) | DTI (FA)  ↓Frontal | DTI (FA)  ↓Corona radiata  ↓Internal capsule | DTI (FA)  - Pons | DTI (FA)  - Cerebellar peduncles |

_↓ Atrophy, for ventricles, indication of enlargement; for MRS, decreased concentration; - No change._

_*Abbreviation: BA, Brodmann area; Cho, Choline; Cr, Creatinine; FA, Fractional anisotropy; GM, Grey matter; MRI, Magnetic resonance imaging; MRS, Magnetic resonance spectroscopy; myo-Ins; myo-Isonitol; NAA, N-Acetylaspartate; SCA, Spinocerebellar ataxia; SMA, Supplementary motor area; VBM, Voxel-based morphometry; WM, White matter._

Adanyeguh, I.M., Henry, P.G., Nguyen, T.M., Rinaldi, D., Jauffret, C., Valabregue, R., Emir, U.E., Deelchand, D.K., Brice, A., and Eberly, L.E. (2015). In vivo neurometabolic profiling in patients with spinocerebellar ataxia types 1, 2, 3, and 7. *Movement Disorders* 30**,** 662-670.

Arruda, W.O., Meira, A.T., Ono, S.E., De Carvalho Neto, A., Betting, L.E.G.G., Raskin, S., Camargo, C.H.F., and Teive, H.a.G. (2020). Volumetric MRI changes in spinocerebellar ataxia (SCA3 and SCA10) patients. *The Cerebellum* 19**,** 536-543.

Bürk, K., Abele, M., Fetter, M., Dichgans, J., Skalej, M., Laccone, F., Didierjean, O., Brice, A., and Klockgether, T. (1996). Autosomal dominant cerebellar ataxia type I clinical features and MRI in families with SCA1, SCA2 and SCA3. *Brain* 119**,** 1497-1505.

Camargos, S.T., Marques-Jr, W., and Santos, A.C.D. (2011). Brain stem and cerebellum volumetric analysis of Machado Joseph disease patients. *Arquivos de Neuro-Psiquiatria* 69**,** 292-296.

Chen, H.-C., Lirng, J.-F., Soong, B.-W., Guo, W.Y., Wu, H.-M., Chen, C.C.-C., and Chang, C.-Y. (2014). The merit of proton magnetic resonance spectroscopy in the longitudinal assessment of spinocerebellar ataxias and multiple system atrophy-cerebellar type. *Cerebellum & ataxias* 1**,** 1-10.

D’abreu, A., França Jr, M.C., Yasuda, C.L., Campos, B.A., Lopes‐Cendes, I., and Cendes, F. (2012). Neocortical atrophy in Machado‐Joseph disease: A longitudinal neuroimaging study. *Journal of Neuroimaging* 22**,** 285-291.

D’abreu, A., França Jr, M.C., Yasuda, C.L., Souza, M.S., Lopes‐Cendes, Í., and Cendes, F. (2011). Thalamic volume and dystonia in Machado–Joseph disease. *Journal of Neuroimaging* 21**,** e91-e93.

De Rezende, T., D'abreu, A., Guimarães, R., Lopes, T., Lopes‐Cendes, I., Cendes, F., Castellano, G., and França Jr, M. (2015). Cerebral cortex involvement in Machado− Joseph disease. *European Journal of Neurology* 22**,** 277-e224.

Etchebehere, E.C., Cendes, F., Lopes-Cendes, I., Pereira, J.A., Lima, M.C., Sansana, C.R., Silva, C.A., Camargo, M.F., Santos, A.O., and Ramos, C.D. (2001). Brain single-photon emission computed tomography and magnetic resonance imaging in Machado-Joseph disease. *Archives of Neurology* 58**,** 1257-1263.

Guimarães, R.P., D'abreu, A., Yasuda, C.L., França Jr, M.C., Silva, B.H., Cappabianco, F.A., Bergo, F.P., Lopes‐Cendes, I.T., and Cendes, F. (2013). A multimodal evaluation of microstructural white matter damage in spinocerebellar ataxia type 3. *Movement Disorders* 28**,** 1125-1132.

Guo, J., Chen, H., Biswal, B.B., Guo, X., Zhang, H., Dai, L., Zhang, Y., Li, L., Fan, Y., and Han, S. (2020). Gray matter atrophy patterns within the cerebellum-neostriatum-cortical network in SCA3. *Neurology* 95**,** e3036-e3044.

Hernandez-Castillo, C.R., Diaz, R., Campos-Romo, A., and Fernandez-Ruiz, J. (2017). Neural correlates of ataxia severity in spinocerebellar ataxia type 3/Machado-Joseph disease. *Cerebellum & Ataxias* 4**,** 1-4.

Inada, B.S.Y., Rezende, T.J.R., Pereira, F.V., Garcia, L.Á.L., Da Rocha, A.J., Neto, P.B., Barsottini, O.G.P., França Jr, M.C., and Pedroso, J.L. (2021). Corticospinal tract involvement in spinocerebellar ataxia type 3: A diffusion tensor imaging study. *Neuroradiology* 63**,** 217-224.

Jao, C.-W., Soong, B.-W., Huang, C.-W., Duan, C.-A., Wu, C.-C., Wu, Y.-T., and Wang, P.-S. (2019a). Diffusion tensor magnetic resonance imaging for differentiating multiple system atrophy cerebellar type and spinocerebellar ataxia type 3. *Brain Sciences* 9**,** 354.

Jao, C.-W., Soong, B.-W., Wang, T.-Y., Wu, H.-M., Lu, C.-F., Wang, P.-S., and Wu, Y.-T. (2019b). Intra-and inter-modular connectivity alterations in the brain structural network of spinocerebellar ataxia type 3. *Entropy* 21**,** 317.

Kang, J.-S., Klein, J., Baudrexel, S., Deichmann, R., Nolte, D., and Hilker, R. (2014). White matter damage is related to ataxia severity in SCA3. *Journal of Neurology* 261**,** 291-299.

Lei, L., Liao, Y., Liao, W., Zhou, J., Yuan, Y., Wang, J., Jiang, H., Shen, L., and Tang, B. (2011). Magnetic resonance spectroscopy of the cerebellum in patients with spinocerebellar ataxia type 3/Machado-Joseph disease. *Zhong nan da xue xue bao. Yi xue ban= Journal of Central South University. Medical Sciences* 36**,** 511-519.

Liang, X., Jiang, H., Chen, C., Zhou, G., Wang, J., Zhang, S., Lei, L., Wang, X., and Tang, B. (2009). The correlation between magnetic resonance imaging features of the brainstem and cerebellum and clinical features of spinocerebellar ataxia 3/Machado-Joseph disease. *Neurology India* 57**,** 578.

Lirng, J.-F., Wang, P.-S., Chen, H.-C., Soong, B.-W., Guo, W.Y., Wu, H.-M., and Chang, C.-Y. (2012). Differences between spinocerebellar ataxias and multiple system atrophy-cerebellar type on proton magnetic resonance spectroscopy. *PLoS One* 7**,** e47925.

Lopes, T.M., Anelyssa, D., Junior, M.C.F., Yasuda, C.L., Betting, L.E., Samara, A.B., Castellano, G., Somazz, J.C., Balthazar, M.L.F., and Lopes-Cendes, I. (2013). Widespread neuronal damage and cognitive dysfunction in spinocerebellar ataxia type 3. *Journal of Neurology* 260**,** 2370-2379.

Meira, A.T., Arruda, W.O., Ono, S.E., Franklin, G.L., De Carvalho Neto, A., Raskin, S., Ashizawa, T., Camargo, C.H.F., and Teive, H.A. (2020). Analysis of diffusion tensor parameters in spinocerebellar ataxia type 3 and type 10 patients. *Parkinsonism & Related Disorders* 78**,** 73-78.

Nunes, M.B., Martinez, A.R.M., Rezende, T.J.R., Friedman, J.H., Lopes-Cendes, I., D'abreu, A., and França Jr, M.C. (2015). Dystonia in Machado–Joseph disease: Clinical profile, therapy and anatomical basis. *Parkinsonism & Related Disorders* 21**,** 1441-1447.

Ogawa, Y., Ito, S., Makino, T., Kanai, K., Arai, K., and Kuwabara, S. (2012). Flattened facial colliculus on magnetic resonance imaging in Machado–Joseph disease. *Movement Disorders* 27**,** 1041-1046.

Peng, H., Liang, X., Long, Z., Chen, Z., Shi, Y., Xia, K., Meng, L., Tang, B., Qiu, R., and Jiang, H. (2019). Gene-related cerebellar neurodegeneration in SCA3/MJD: A case-controlled imaging-genetic study. *Frontiers in Neurology* 10**,** 1025.

Rezende, T.J.R., De Paiva, J.L.R., Martinez, A.R.M., Lopes‐Cendes, I., Pedroso, J.L., Barsottini, O.G.P., Cendes, F., and França Jr, M.C. (2018). Structural signature of SCA3: From presymptomatic to late disease stages. *Annals of Neurology* 84**,** 401-408.

Schulz, J.B., Borkert, J., Wolf, S., Schmitz-Hübsch, T., Rakowicz, M., Mariotti, C., Schoels, L., Timmann, D., Van De Warrenburg, B., and Dürr, A. (2010). Visualization, quantification and correlation of brain atrophy with clinical symptoms in spinocerebellar ataxia types 1, 3 and 6. *Neuroimage* 49**,** 158-168.

Wang, P.-S., Chen, H.-C., Wu, H.-M., Lirng, J.-F., Wu, Y.-T., and Soong, B.-W. (2012). Association between proton magnetic resonance spectroscopy measurements and CAG repeat number in patients with spinocerebellar ataxias 2, 3, or 6. *PLoS One* 7**,** e47479.

Wu, X., Liao, X., Zhan, Y., Cheng, C., Shen, W., Huang, M., Zhou, Z., Wang, Z., Qiu, Z., and Xing, W. (2017). Microstructural alterations in asymptomatic and symptomatic Patients with spinocerebellar ataxia Type 3: A tract-based spatial statistics study. *Frontiers in Neurology* 8**,** 714-722.

Yoshizawa, T., Watanabe, M., Frusho, K., and Shoji, S. (2003). Magnetic resonance imaging demonstrates differential atrophy of pontine base and tegmentum in Machado–Joseph disease. *Journal of the Neurological Sciences* 215**,** 45-50.
